# Insulin Hot-Spot Analogs Formed with *N*-Methylated Amino Acid Residues Inhibit Aggregation of Native Hormone

**DOI:** 10.3390/molecules24203706

**Published:** 2019-10-15

**Authors:** Monika Swiontek, Joanna Wasko, Justyna Fraczyk, Krystian Galecki, Zbigniew J. Kaminski, Beata Kolesinska

**Affiliations:** 1 Institute of Organic Chemistry, Faculty of Chemistry, Lodz University of Technology, Zeromskiego 116, 90-924 Lodz, Poland; monika.swiontek@gmail.com (M.S.); joanna.wasko@edu.p.lodz.pl (J.W.); justyna.fraczyk@p.lodz.pl (J.F.); zbigniew.kaminski@p.lodz.pl (Z.J.K.); 2 Institute of General Food Chemistry, Faculty of Biotechnology & Food Sciences, Lodz University of Technology, Stefanowskiego 4/10, 90-924 Lodz, Poland; krystian.galecki@p.lodz.pl

**Keywords:** inhibition of insulin aggregation, aggregation, *N*-methylated analogs of insulin hot-spots, microwave-assisted solid-phase peptide synthesis, triazine coupling reagent

## Abstract

In this study, *N*-methylated analogs of hot-spots of insulin were designed and synthesized, in the expectation that they would inhibit the aggregation of both insulin hot-spots and the entire hormone. Synthesis of insulin “amyloidogenic” analogs containing *N*-methylated amino acid residues was performed by microwave-assisted solid phase according to the Fmoc/tert-Bu strategy. As a coupling reagent 4-(4,6-dimethoxy-1,3,5-triazin-2-yl)-4-methylmorpholinium toluene-4-sulfonate (DMT/NMM/TosO^-^) was used. Three independent methods were applied in aggregation studies of the complexes of insulin with its *N*-methylated peptides. Additionally, circular dichroism (CD) measurements were used to confirm that aggregation processes did not occur in the presence of the *N*-methylated analogs of hot-spot insulin fragments, and that insulin retains its native conformation. Of the seven *N*-methylated analogs of the A- and B-chain hot-spots of insulin, six inhibited insulin aggregation (peptides **1** and **3**–**7**). All tested peptides were found to have a lower ability to inhibit the aggregation of insulin hot-spots compared to the capability to inhibit native hormone aggregation.

## 1. Introduction

Insulin, a hormone that regulates the metabolism of carbohydrates, aggregates to form insoluble fibrillar structures in vitro and in vivo. In solution, aggregation of insulin is driven by elevated temperatures, acidic pH, and solution ionic strength [1]. Aggregation can dramatically affect the subcutaneous bioavailability and pharmacokinetic of insulin, and in extreme cases totally prevent absorption. Moreover, formation of insoluble amyloid structure by insulin is a big limitation when it comes to the long-term storage of insulin [2].

One of the first research works shows that insulin fibrils are built from the globular state of insulin molecules [3]. Based on results obtained using several complementary research techniques (including X-ray diffraction, and scanning-transmission electron microscopy), Ivanova et al. proposed a structure of insulin amyloid fibrils [4]. The amyloidogenic core of insulin consists of a repeated peptide motif, which is known as steric zipper and is formed by two peptide fragments B12–B17 H-LeuValGluAlaLeuTyrLeu-OH (or B13–B17 H-ValGluAlaLeuTyrLeu-OH), while fragment A12–A19 H-SerLeuTyrGlnLeuGluAsnTyr-OH (or A13–A19 H-LeuTyrGlnLeuGluAsnTyr-OH) stabilize structure of steric zipper. Stabilization of the formed fibrils spines is driven by interactions between Tyr residue in the second position from fragment A13–A19 and Tyr from B11–B17. This interaction has been termed “kissing tyrosine” and it is characteristic for the wet interface of crystalline H-LeuValGluAlaLeuTyrLeu-OH [4]. Surin et al. found that the amyloidogenic regions of insulin: A-chain (A8–A15) and B-chain (B12–B19 and B23–B27) predicted by using FoldAmyloid, Waltz, and AGGRESCAN overlapped fragments A4–A20 and B5–B25 formed under limited proteolysis [5].

The process of insulin fibrillation is inhibited by molecules that either avert folding of the hormone native protein or insulate incompletely folded aggregates. Protein aggregation can be inhibited by short modified or not modified peptides, which possess an ability to interfere with β-sheet structures, resulting in changes in their geometry and prevents attachment of subsequent components of the growing aggregate. These peptides are termed as self-recognitions domains and their amino acids composition is usually homological to corresponding hot-spots structure. A nine-residue peptide, NK9 (NIVNVSLVK), has been found to delay the fibrillation of insulin, even in a sub-stoichiometric ratio [6]. To determine the step of insulin fibrillation that NK9 affects, Banerjee et al. performed a study in which insulin was incubated with NK9 for different lengths of time (0, 15, 20, and 30 min) [6]. The maximal elongation time for the early nucleation step, 200 min, was achieved when NK9 was added at the start of incubation. The addition of NK9 after 15 min of incubation reduced the nucleation process to 190 min. The strongest effect was therefore observed when NK9 was added at the beginning of incubation. It was also found that adding NK9 after 30 min of incubation did not reduce the nucleation time, but influenced the morphology of the fibrils, indicating interaction between NK9 and the protofibrils. The number of β-sheets in the sample with inhibitor NK9 was reduced by about 60%.

Surprisingly, several studies have also used peptides consisting only of arginine residues to suppress protein aggregation [7,8,9,10,11,12,13]. Arginine at 100–200 mM has been found to significantly inhibit process of insulin aggregation, resulting lag time was 75 h [14]. Another strategy to reduce insulin aggregation is to use small hybrid peptides, consisting of a recognition domain designed specifically to bind to insulin and a disrupting domain that alters insulin aggregation. This strategy acts directly on the selected “amyloidgenic core” of insulin and typically uses short peptides, which are involved in the recognition process and thus are responsible for triggering aggregation. It has been suggested that fragment B11-B18 LVEALYLV is involved in insulin misfolding and aggregation, as well as being a building block in the insulin spine [15]. Conjugate RRRRRRLVEALYL, even at a concentration of 2 mM, has been found to inhibit insulin aggregation significantly, although not completely. However, monomeric forms of insulin were not detected in the solution. The inhibitory activity of an analog with R6 hexamer attached to the LVEALYL C-terminus, LVEALYLRRRRRR, has also been investigated. Again, it was observed that the formation of fibrils was inhibited, but the impact was not as great as in the case of conjugate RRRRRRLVEALYL. Moreover, the introduction of arginine residues into the amyloidogenic core of insulin reduced the concentration of aggregation inhibitors around 100-fold.

The embedding of *N*-methyl amino acids into peptide chains to produce inhibitors of amyloidosis has attracted considerable attention. As in the aforementioned strategy, the peptides correspond to a region crucial to the aggregation process. Incorporation of *N*-methyl amino acids results in the inability to form the intermolecular hydrogen bond responsible for stabilization of fibrillar structure. Moreover, steric hindrance impedes approaching subsequent monomers. The best inhibitory effect was observed, when *N*-methyl amino acids were placed in alternating positions of the sequence. This approach was successfully implemented to inhibit Aβ40 fibrillogenesis [16,17,18]. Such designed inhibitors also possess additional advantages of high proteolytic resistance, solubility, blood-brain barrier permeability, and the propensity to form β-structures at the *N*-methylated site [19]. Kapurniotu et al. shown that the introduction of *N*-alkylated amino acid residues into the 20-27 human amylin fragment resulted in the inhibition of β-sheet rich fibrils formation. [20].

Another approach to impede the aggregation of proteins/peptides relies on reducing the predisposition of β-sheet formation. Replacing the L-α-amino acids in a peptide chain with α,α-disubstituted amino acid forces the formation of a stable right-handed (P) helical structure, modifying conformation of the peptide chain which triggers the formation of stable helical structures [21]. However, there are no reports in the literature on the use of either *N*-ethylated peptides or α,α-disubstituted amino acid to prevent insulin aggregation.

The present study set out to investigate the possibility of using analogs of insulin fragments which are crucial in the aggregation process to inhibit the process of insulin fibrillization. The aim of the study was to design and synthesize *N*-methylated fragments of the native hormone, which would then be evaluated for their ability to inhibit aggregation of the hormone. Analogs of the two native hormone fragments were used, which will be referred to as hot-spot A (A13–A19) and hot-spot B (B12–B17) respectively. Residues of *N*-methylated amino acids were incorporated into both. We expected that the presence of a methyl group on the nitrogen atom of the peptide bond would destabilize the aggregates, as a result of the inability of neighboring peptide chains to form hydrogen bonds (the lack of hydrogen in the peptide bond), as well as due to the steric hindrance of the methyl group.

## 2. Results and Discussion

When designing the analogs for hot-spots A (A13–A19) and B (B12–B17), it was assumed that one or two residues of the *N*-methylated amino acids would be incorporated into the peptide. It was further assumed that the modification would not be subjected to the residue of the *N*-terminal amino acids, since the removal of one hydrogen atom from the NH_2_ group would not prevent the formation of a hydrogen bond with the CONH group of the neighboring peptide chain. Since the synthesis of both aggregating peptides and their analogs containing residues of *N*-methylated amino acids poses a significant technical challenge, a solid-phase procedure supported by microwave radiation was employed. As a coupling reagent, 4-(4,6-dimethoxy-1,3,5-triazin-2-yl)-4-methylmorpholinium toluene-4-sulfonate (DMT/NMM/TosO-) was used, which has documented effectiveness for the synthesis of difficult peptides both in solution and in the solid phase [22]. The following analogs of hot-spots A and B of insulin were designed and synthesized: H-Leu(N-Me)TyrGlnLeuGluAsnTyr-OH (**1**), H-LeuTyrGln(N-Me)LeuGluAsnTyr-OH (**2**), H-Leu(N-Me)TyrGln(N-Me)LeuGluAsnTyr-OH (**3**), H-ValGluAla(N-Me)LeuTyrLeu-OH (**4**), H-ValGlu(N-Me)AlaLeuTyrLeu-OH (**5**), H-ValGluAlaLeu(N-Me)TyrLeu-OH (**6**), and H-ValGlu(N-Me)AlaLeu(N-Me)TyrLeu-OH (**7**). The purity of the crude products obtained by classic microwave-assisted solid-phase peptide synthesis was between 91 and 98% (see Supporting Materials) according to LC-MS measurements.

### 2.1. Predisposition of Peptides 1–7 to Aggregate

The first aim of the study was investigation on predisposition of peptides **1**–**7** to aggregate. Native fragments of insulin (hot-spots A and B) were used as an internal control [23]. To mimic natural conditions inside the human body, the aggregation of peptides was carried out at pH 7.2 phosphate buffer in 37 °C. The aggregation process leads to the formation of ordered insoluble fibrils. Therefore, three independent tests were used to assess the formed aggregate. To determine aggregation propensity analogs **1–7** were examined by three independent aggregation tests, which are nonspecific for amyloids but recommended [24,25,26,27,28,29,30,31,32]: the Congo Red (CR) test, the Thioflavin T (ThT) assay, and microscopic visualization of samples stained with CR. The CR test showed a significant shift in the maximum absorption characteristic for the aggregates. A positive ThT assay resulted in a significant increase in the fluorescence intensity of the indicator in the presence of aggregates in comparison to the control (ThT only). Both with and without polarizing filters, microscopic examination of the samples stained with CR revealed fibrous structures.

**Figure 1:**
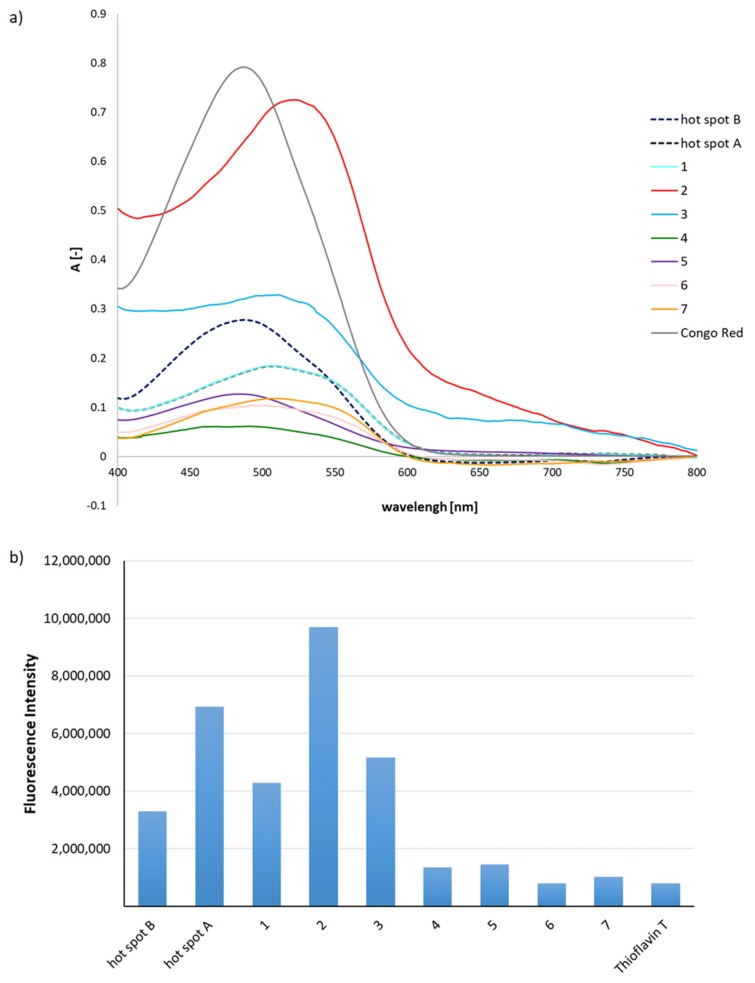

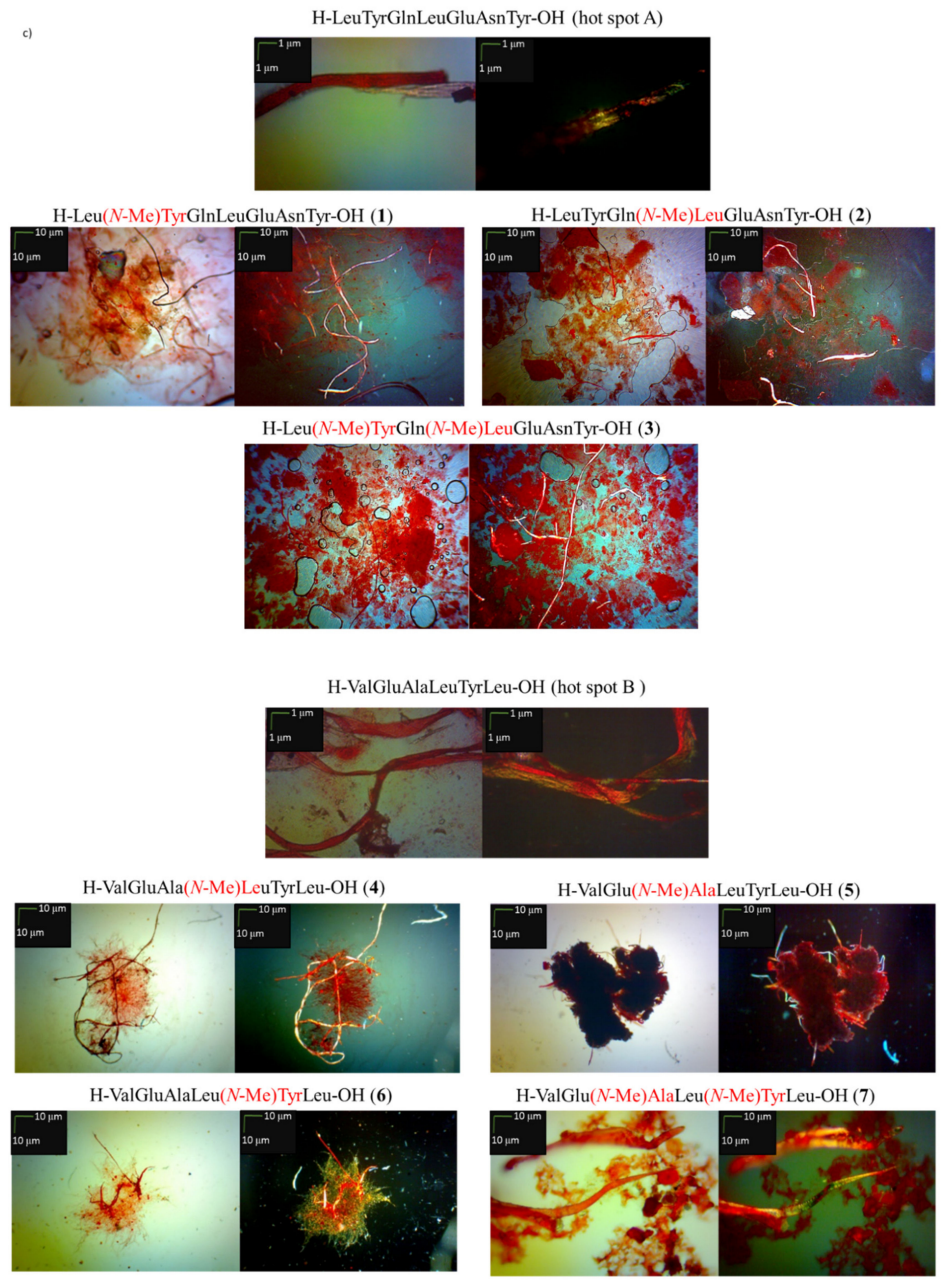
(**a**) Ultraviolet (UV) spectra of peptides **1**–**7** incubated with Congo Red (CR). Results derived after the fourth day of incubation; (**b**) fluorescence intensity spectra of peptides **1**–**7** in the presence of Thioflavin T (ThT), wavelength = 485 nm, after fourth day of incubation; (**c**) pictures of **1**–**7** obtained without a polarized filter (left side) and with a polarized filter (right side). Scale bars, 10 µm. Microscopic measurements have been performed on the fourth day of incubation.

The hot-spot A analogs, peptides **1**–**3**, revealed a decrease in the absorbance value and a shifting of the maximum absorbance to 534–541 nm in all cases (Figure 1a). The influence of the *N*-methylated amino acid residues was varied in peptides **1**–**3**, as shown by fluorescence studies (ThT assay) (Figure 1b). Reductions in the fluorescence intensity to 40% for H-Leu(*N*-Me)TyrGlnLeuGluAsnTyr-OH (**1**) and 25% for H-Leu(*N*-Me)TyrGln(*N*-Me)LeuGluAsnTyr-OH (**3**) were observed, compared to native hot-spot A. The ThT assay score for H-LeuTyrGln(*N*-Me)LeuGluAsnTyr-OH (**2**) was rather surprising. Significantly greater susceptibility to aggregation was found compared to the native fragment. The data obtained indicate that the tyrosine residue plays a significant role stabilizing aggregates, but not because of its effect on hydrogen bonding within the phenol group (kissing tyrosine) [4,33]. Instead, it is due to the ability of hydrogen bonds involving the tyrosine NH group. The introduction of a methyl substituent to this group in peptides **1** and **3** resulted in a significant reduction in their susceptibility to aggregate.

The CR assay results for *N*-methylated analogs **4**–**7** of hot-spot B indicated diversity in their susceptibility to aggregate (Figure 1a). Peptides H-ValGluAla(*N*-Me)LeuTyrLeu-OH (**4**) and H-ValGlu(*N*-Me)AlaLeuTyrLeu-OH (**5**) showed no characteristic shift in maximum absorbance, although the absorbance value was very low, possibly due to the lack of interaction between the dye and the peptides. This result indicates very low susceptibility to aggregate, what may be useful for inhibiting the aggregation process. For peptides H-ValGluAlaLeu(*N*-Me)TyrLeu-OH (**6**) and H-ValGlu(*N*-Me)AlaLeu(*N*-Me)TyrLeu-OH (**7**), maximum absorbance has been shifted to 537 nm, what proved the formation of a complex between the fibrous aggregate and CR, and therefore provided evidence of aggregation. The ThT assay clearly indicated that peptides **4**–**7** do not have the ability to aggregate. Their fluorescence intensity value was 70% lower than that of the native hot-spot B (Figure 1b). Microscopic examinations (Figure 1c) of CR-stained samples indicated the presence of amorphous structures, in which golden birefringence (fibrous structures characterized by polarized light) was also visible. Peptides **1**–**3** were characterized by significant susceptibility to aggregate, based on the results of CR and ThT assays. However, the microscopic photographs show a surprisingly small proportion of fibrous structures in comparison to the amorphous form. A reverse relationship was found for peptides **6** and **7**, which appeared to be resistant to aggregation based on dye tests, while microscopic studies revealed a significant proportion of fibrous structures, especially in the case of H-ValGlu(*N*-Me)AlaLeu(*N*-Me)TyrLeu-OH (**7**).

### 2.2. Effects of Peptides 1–7 on the Aggregation of Native Insulin Hot-Spots A and B

**Figure 2:**
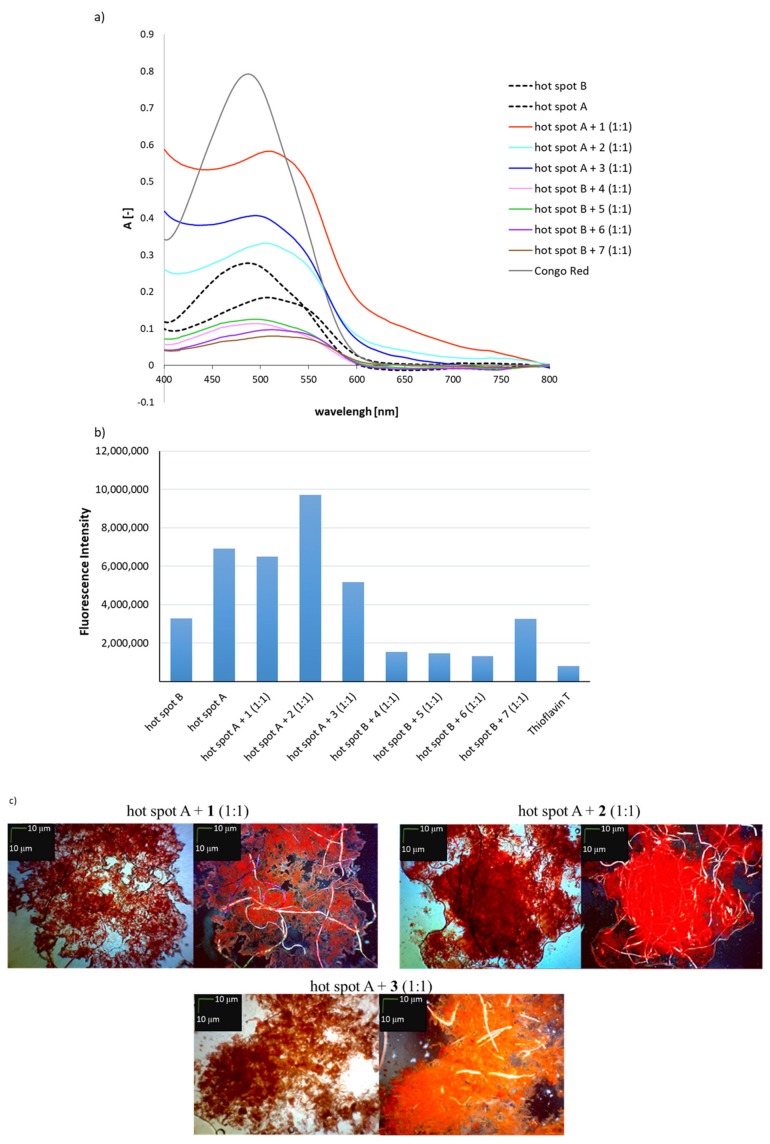

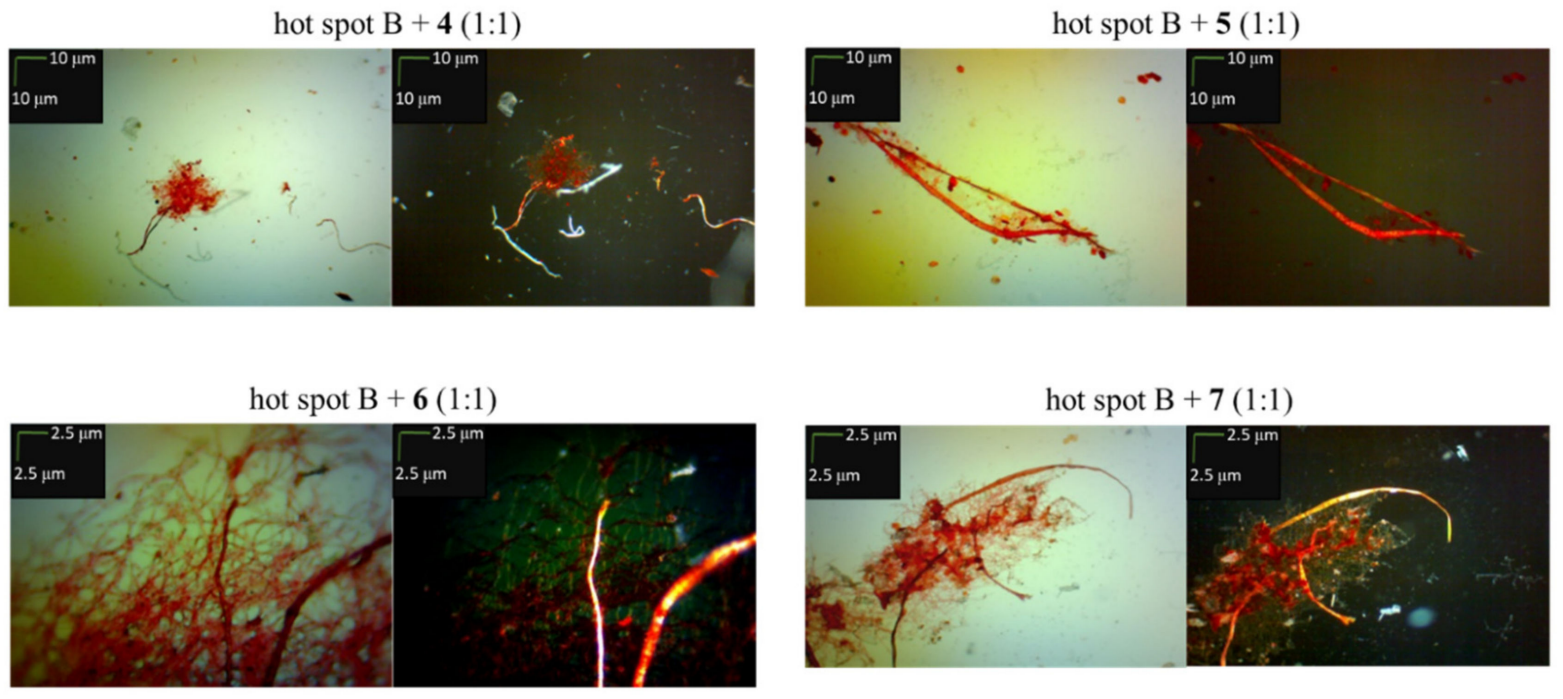
(**a**) UV spectra of mixtures (1:1) of hot-spot A with one of the peptides **1**–**3** and hot-spot B with one of the peptides **4**–**7** in the presence of Congo Red (CR). The spectra show results obtained on the fourth day of incubation; (**b**) fluorescence intensity spectra of mixtures (1:1) of hot-spot A with one of the peptides **1**–**3** and hot-spot B with one of the peptides **4**–**7** in the presence of Thioflavin T (ThT), wavelength = 485 nm, fourth day of incubation; (**c**) pictures of mixtures (1:1) of hot-spot A with one of the peptides **1**–**3** and hot-spot B with one of the peptides **4**–**7**, without a polarized filter (left side) and with a polarized filter (right side). Scale bars, 10 µm. In all cases, the samples were taken for microscopic examination on the fourth day of incubation.

Peptides **1**–**7** were further studied to discover whether they would inhibit the aggregation of native insulin hot-spots A and B. Aggregation experiments were conducted under physiological conditions in equimolar (1:1) mixtures of the hot-spots and their *N*-methylated analogs. Hot-spot A was incubated with peptides **1**–**3** and hot-spot B with peptides **4**–**7** (Figure 2). For the mixtures of hot-spot A with peptides **1**–**3**, the UV-Vis spectra (CR assay) show a shift of the absorption maximum at 521 nm in all cases. However, it was found that the absorbance value was much higher compared to the native peptide (Figure 2a). For the mixtures of hot-spot B with *N*-methylated analogs (peptides **4** and **5**), no shift in the maximum absorbance was observed, which indicated a lack of aggregation. For the equimolar mixtures with peptides **6** and **7**, shifts in the maximum absorbance to about 550 nm, characteristic for aggregates, were observed. A significant decrease in the absorbance value was observed in the CR tests for all mixtures of hots spot B with its *N*-methyl analogs.

The results of a ThT assay performed on equimolar mixtures of hot-spot A with peptides **1**–**3** indicate that only when peptide **3** was used, the fluorescence intensity was reduced by 25% compared to native hot-spot A (Figure 2b). The mixture of hot-spot A with peptide **2** resulted with significant increase in fluorescence intensity, which means that the complex had higher aggregation susceptibility compared to fragment A13–A19.

**Figure 3:**
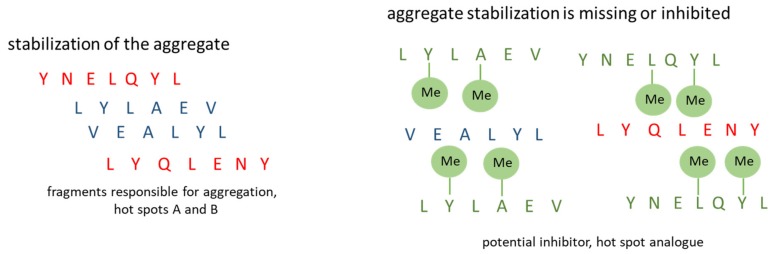
Model of a 1:2 complex consisting of one hot-spot molecule and two molecules of *N*-methylated analog.

For equimolecular mixtures of hot-spot B with peptides **4**–**7**, it was found that only in the case of the complex of hot-spot B with H-ValGlu(*N*-Me)AlaLeu(*N*-Me)TyrLeu-OH (**7**) the fluorescence intensity was not reduced compared to the native B12–B17 fragment (Figure 2b). For peptides **4**, **5**, **6** containing single residues of *N*-methylated amino acids in the peptide chain, a 60% decrease in fluorescence intensity was observed. This result indicates that these peptides inhibit the aggregation of the native hot-spot B (fragment B12–B17 of insulin). Microscopic examination revealed amorphous structures interwoven with fibrous structures, which suggests that aggregation was incomplete (Figure 2c). For the complexes of hot-spot B with peptides **6** or **7**, characteristic golden birefringence was observed under polarized light. Attempts were also made to increase the number of *N*-methylated analogs of hot-spots of insulin, in the expectation that 1:2 complexes composed of one hot-spot molecule and two molecules of *N*-methylated analog would form sandwich structures, and thus inhibit the aggregation of the native fragments (Figure 3).

**Figure 4:**
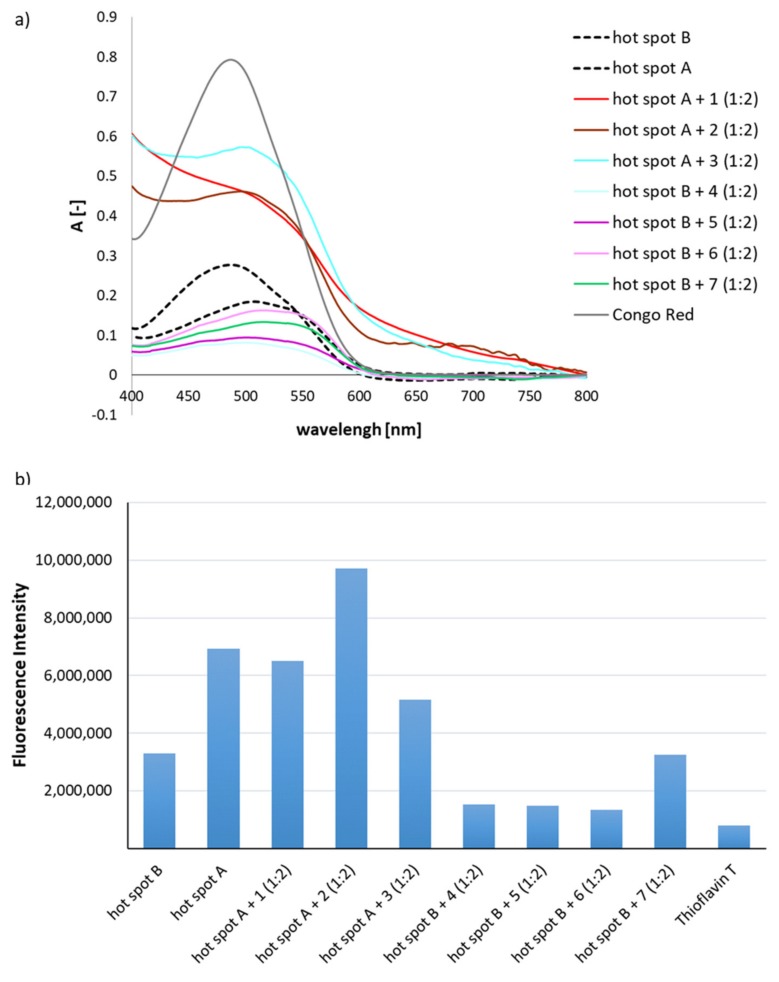

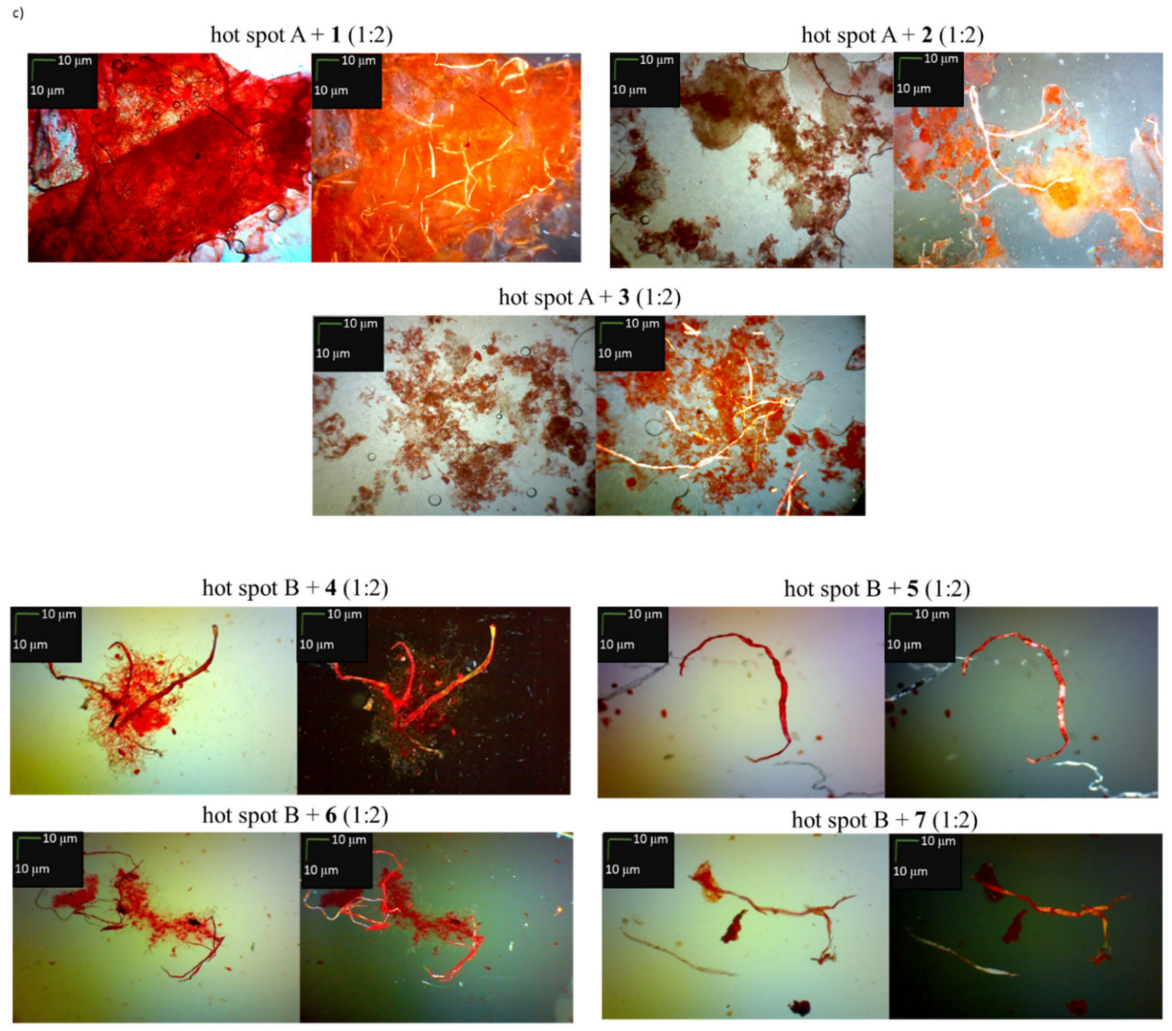
(**a**) UV spectra of mixtures (1:2) of hot-spot A with one of the peptides **1**–**3** and hot-spot B with one of the peptides **4**–**7** in the presence of Congo Red (CR). The spectra show results obtained on the fourth day of incubation; (**b**) fluorescence intensity spectra of mixtures (1:2) of hot-spot A with one of the peptides **1**–**3** and hot-spot B with one of the peptides **4**–**7** in the presence of Thioflavin T (ThT), wavelength = 485 nm, fourth day of incubation; (**c**) pictures of mixtures (1:2) of hot-spot A with one of the peptides **1**–**3** and hot-spot B with one of the peptides **4**–**7**, without a polarized filter (left side), with a polarized filter (right side). Scale bars, 10 µm. In all cases, the samples were taken for microscopic examination on the fourth day of incubation.

However, the results of increasing the concentration of potential aggregation inhibitors were ambiguous. The results of the CR assay (Figure 4a) and ThT assay (Figure 4b) were very similar to those obtained for equimolar 1:1 complexes of hot-spot A with peptide **1**–**3** or hot-spot B with peptides **4**–**7** (Figure 2a,b). On the other hand, microscopic studies of hot-spots A and B with increased content of peptides **1**–**7** (1:2 complexes) (Figure 4c) revealed that amorphous structures were dominant, indicating the inhibition of native hot-spot aggregation.

Nevertheless, in all cases the examined complexes also contained fibrous structures (aggregates). Thus, peptides **1**–**7** reduced the aggregation of A and B hot-spots, but in no instance was aggregation completely inhibited.

### 2.3. Effects on Aggregation of Human Insulin

**Figure 5:**
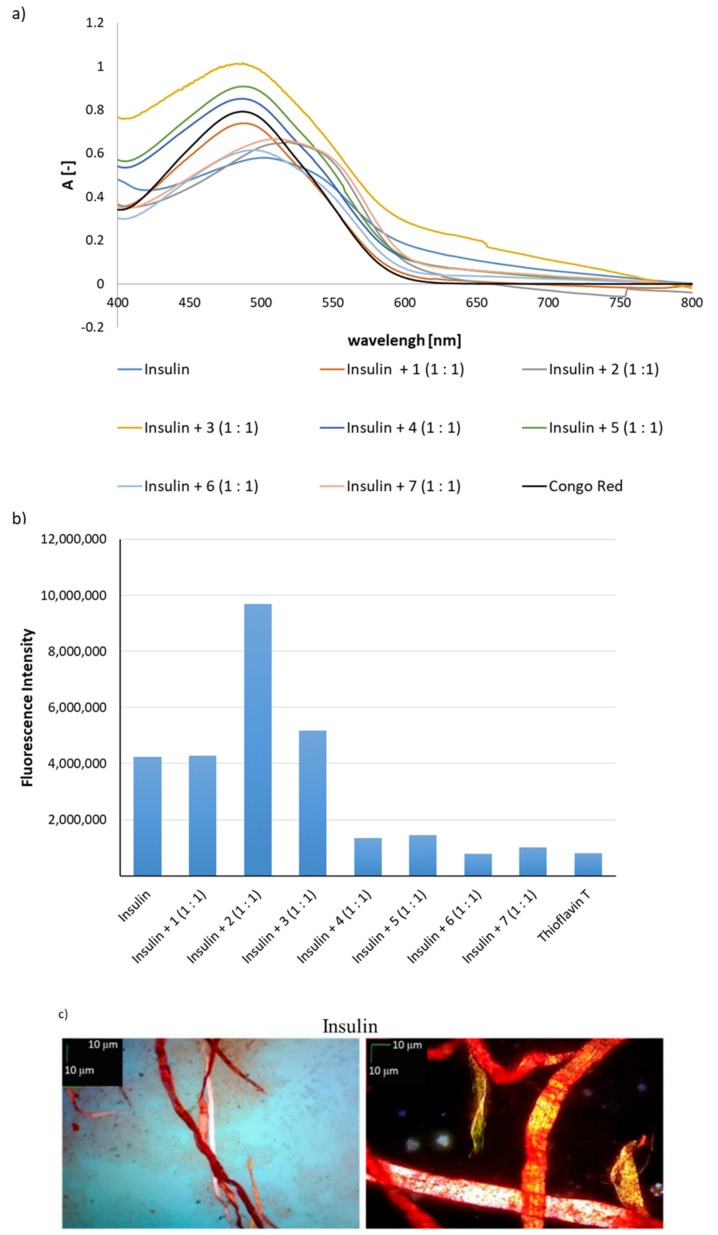

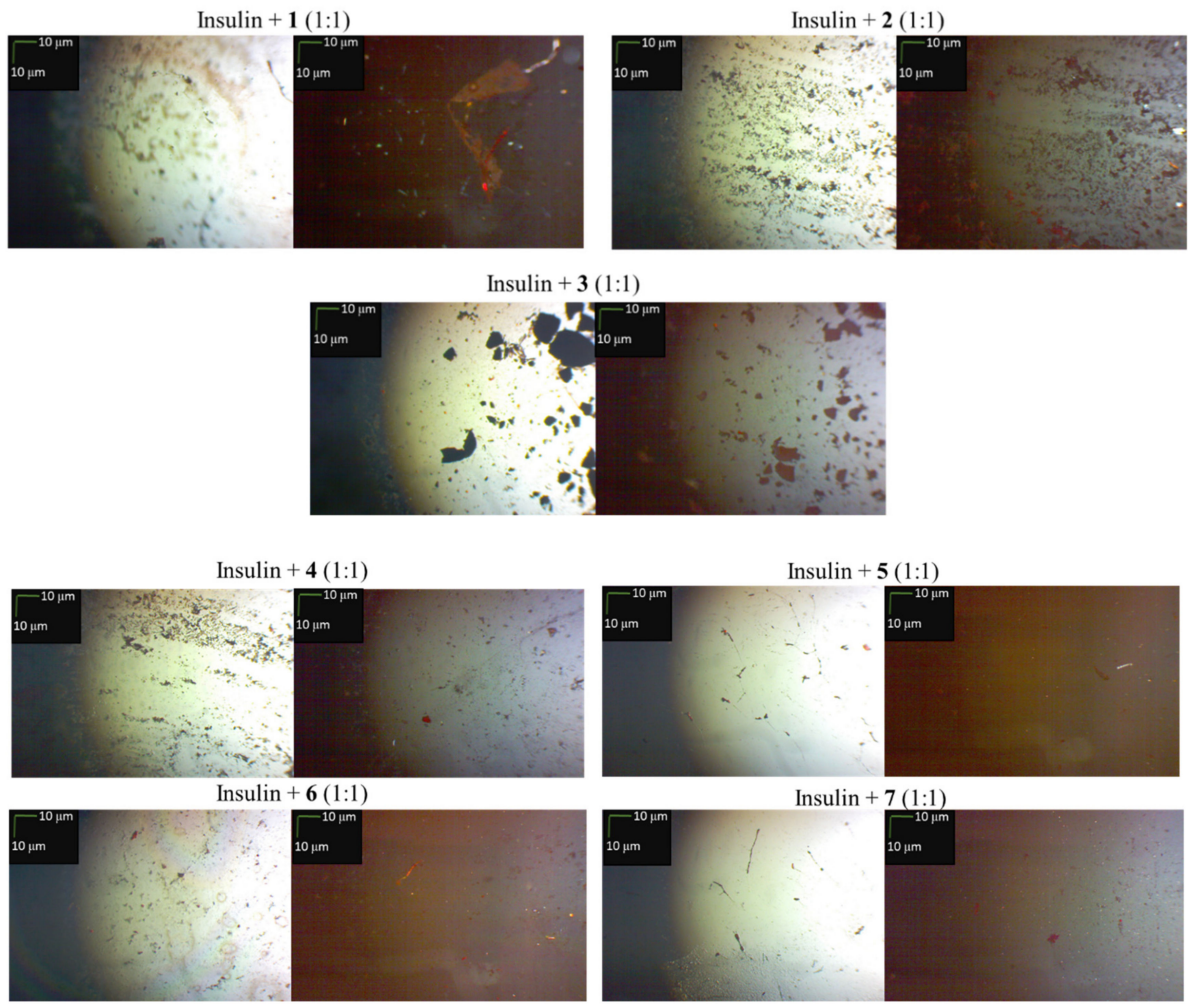
(**a**) UV spectra of mixtures (1:1) of insulin with one of the peptides **1**–**7** in the presence of Congo Red (CR). Results derived on the fourth day of incubation; (**b**) fluorescence intensity spectra of mixtures (1:1) of insulin with one of the peptides **1**–**7** in the presence of Thioflavin T (ThT), wavelength = 485 nm, after fourth day of incubation; (**c**) pictures of mixtures (1:1) of insulin with one of the peptides **1**–**7**, without a polarized filter (left side), with a polarized filter (right side). Scale bars, 10 µm. Microscopic measurements have been performed on the fourth day of incubation.

In the final stage of the study, an attempt was made to determine whether peptides **1**–**7** would inhibit the aggregation of human insulin. Zinc-free insulin was used for the study. The same methods of analysis were used as previously for hot-spots A and B. Peptides **1**–**7** were used in stoichiometric amounts relative to the native hormone (complex 1:1). Equimolar mixtures of insulin with each of the peptides **1**–**7**, before adding the appropriate dye, were preincubated for 7 days at 37 °C. The results of a CR test showed the largest shifts of the maximum absorbance in relation to the CR spectrum in the cases of peptides **2** and **7**, demonstrating the aggregation of a complex of insulin with a potential inhibitor. The maximum absorbance shifts were 540 nm and 560 nm, respectively. In other cases, when peptides **1**, **3**, **4**, **5**, **6** were used, a slight shift of the CR absorbance peak was observed (Figure 5a). Thus, based on CR assay results, it can be concluded that peptides **1**, **3**, **4**, **5**, **6** inhibit insulin aggregation.

However, the results of a ThT assay (Figure 5b) showed that peptides **1** and **3** did not affect the susceptibility of insulin to aggregate (the fluorescence intensity was comparable to that obtained for insulin alone). The result for peptide **2** was surprising. A two-fold increase in fluorescence intensity was observed, which may indicate that the complex peptide **2**–insulin has significantly higher aggregation ability than its individual components. The presence of peptides **4**–**7**, which are hot-spot B analogs, significantly reduced and even completely inhibited the ability of insulin to aggregate. The fluorescence intensity values were comparable to the fluorescence intensity of Thioflavin T. The third examination test based on microscopic studies showed that all peptides **1**–**7** derived from insulin hot-spots A and B all inhibited the ability of insulin to aggregate (Figure 5c). Microscopic tests both without and with the use of a polarizing filter showed no aggregates. A summary of the inhibitory properties of peptides **1**–**7** is presented in Table 1.

**Figure 6:**
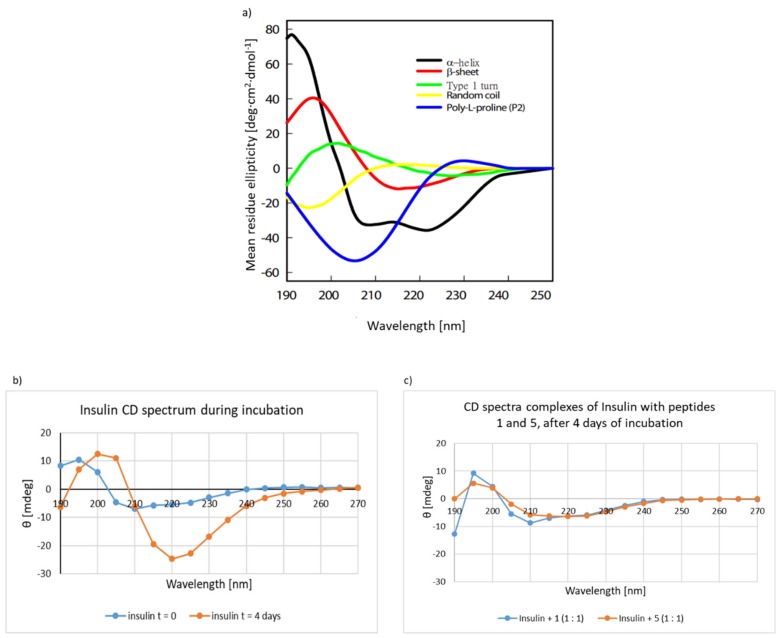
CD spectra. (**a**) model protein with stable secondary structure, (**b**) insulin, (**c**) complexes of insulin with peptides **1** and **5**. The peptides and complexes were incubated in phosphate buffer solution, pH 7.2, concentration 0.1 mg/mL at 37 °C, t = 0 (CD spectrum of insulin after dissolving the sample); t = 4 days (CD spectrum of peptides after 4 days of incubation).

Having established the ability of peptides **1**–**7** to prevent insulin aggregation using three independent tests, attempts were made to confirm this observation using the circular dichroism (CD) technique for two selected inhibitors of aggregation by peptides **1** and **5** derived from hot-spots A and B. We expected that the CD spectra of the insulin–peptide complexes would be identical or close to the native insulin curve. CD measurements were performed on freshly prepared insulin samples in phosphate buffer solution, pH 7.2, at 0.1 mg/mL at t = 0 (Figure 6b) and after 4 days of incubation (Figure 6b). Comparing the obtained curves with the spectra of model proteins with a strictly defined secondary structure (Figure 6a) reveals that the spectrum recorded at t = 0 corresponds to the α-helix structure and the spectrum registered after 4 days of incubation most closely resembles the spectrum characteristic for β-sheet proteins. Not complete matching (reflection) may be because insulin aggregation was carried out only for 4 days at 37 °C in buffer at pH 7.2.

Interpretation of CD spectra, especially in the case of aggregating peptides, is challenging (Figure 6a). Disordered peptides show a low ellipticity at 210 nm and negative band at 195 nm. Peptides with well-defined β-helices (antiparallel β-sheet) possess negative band at 218 nm and positive band at 195 nm [34,35,36]. Typical shifts occur when random coil conformation changes via the α-helix to amyloid fibrils. The situation becomes even more complicated in the initial phase of aggregation because the amorphous aggregates contain mainly α structures [37] which are transformed into mature amyloid fibrils. Additionally, diversity assigned to β-structures, which refer to different orientation and twisting, turning of β-sheets cause misinterpretation of CD spectra. This is due to the fact that those structures are forming β-helices, which often possess similar negative bands that normally are assigned to α-helix conformation (negative bands at 222 nm and 208 nm, a positive band at 193 nm) [38,39]. Given the complexity of the problem, we performed CD studies of the complexes insulin–peptide **1** and peptide **5** in a 1:1 ratio (Figure 6c). The complexes were incubated for 4 days in phosphate buffer at pH 7.2. The obtained spectra were found to have a specific fingerprint characteristic of the native insulin structure (t = 0), which confirms the results of CR, ThT, and microscopic tests. In each case, the spectra obtained did not reflect the CD spectrum of the aggregated insulin (Figure 6b).

## 3. Materials and Methods

The peptides H-Leu(*N*-Me)TyrGlnLeuGluAsnTyr-OH (**1**), H-LeuTyrGln(*N*-Me)LeuGluAsnTyr-OH (**2**), H-Leu(*N*-Me)TyrGln(*N*-Me)LeuGluAsnTyr-OH (**3**), H-ValGluAla(*N*-Me)LeuTyrLeu-OH (**4**), H-ValGlu(*N*-Me)AlaLeuTyrLeu-OH (**5**), H-ValGluAlaLeu(*N*-Me)TyrLeu-OH (**6**), and H-ValGlu(*N*-Me)AlaLeu(*N*-Me)TyrLeu-OH (**7**) were synthesized in a Liberty Blue (CEM, Matthews, NC, USA) automatic peptide synthesizer, using microwave irradiation and standard Fmoc solid-phase synthesis. Fmoc-protected amino acids were purchased from Novabiochem (San Diego, CA, USA) or Bachem AG (Bubendorf, Switzerland). Human insulin without zinc was purchased from Sigma-Aldrich (Saint Louis, MO, USA). Deprotection steps were performed at 75 °C for 3 min. Coupling steps were performed at 75 °C for 5 min. The system was equipped with an inert gas option for dispersing resin inside the reactor. The resin used for all peptides was 2-chlorotrityl chloride. As a coupling DMT/NMM/TosO^-^ was used, prepared according to a procedure described previously [22].

Analytical reverse-phase high-performance liquid chromatography (RP-HPLC) was performed on a Waters HPLC (Waters Corporation, Milford, MA, USA) system, equipped with Vydac C18 column (10 cm × 4.6 mm) with a flow rate of 0.4 mL/min, eluting with 0.1% TFA in H_2_O (B) and 0.1% TFA in CH_3_CN (A). All HPLC spectra of crude **1**–**7** peptides are available in Supplementary Materials: Figures S1, S3, S5, S7, S9, S11, and S13.

Electron ionization mass spectrometry ESI-MS was carried out on a Bruker microOTOF-QIII (Bruker Corporation, Billerica, MA, USA). MS spectra of **1**–**7** peptides are available in Supplementary Materials: Figures S2, S4, S6, S8, S10, S12, and S14.

Preparative HPLC. Performed on a CombiFlash, EZPrep, Teledyne ISCO (Lincoln, Nebraska, USA) using a Supelco Discovery BIO Wide Pore C18 column (25 cm × 21.2 mm, 10 mm; Sigma-Aldrich); flow rate, 5 mL/min; detection wavelengths, 220 and 254 nm), gradient ratio A (0.1% TFA in MeCN) and B (0.1% TFA in H_2_O) 0:100 to 18:82 in 30 min and initial isocratic period of 5 min.

CD studies were carried out on Jasco J-1500 spectrometer (ABLE-JASCO Polska, Cracow, Poland). Far-UV CD experiments were performed using a J-1500 CD spectrometer (Jasco). All samples were prepared by addition of phosphate buffer pH 7.2 (10 mL) to 1 mg of analyzed peptide, insulin, and peptide–insulin complexes; final concentration was 0.1 mg/mL. All measurements were carried out in room temperature in quartz cuvette (1 mm path length, Hellma). Other experimental settings were as follows: measurements range: 190–270 nm, data pitch, 5 nm; scanning mode, continuous; scanning speed, 100 nm/min; bandwidth, 3 nm; integration time, 1 s.

### 3.1. General Methods for Solid-Phase Peptide Synthesis

#### 3.1.1. Method 1. Resin Loading

At room temperature, 1 g of 2-chlorotrityl chloride resin (CTC) with maximum loading of 1 mmol/g was preswollen for 30 min in dry dichloromethane (DCM). Subsequently, 3 eq. of the appropriate Fmoc-protected amino acid was dissolved in DCM, to which 6 eq. *N*, *N*-diisopropylethylamine (DIPEA) was added. This loading solution was added to 2-chlorotrityl chloride resin and the suspension was shaken for 2 h. After this time, three washings steps with DCM were performed. To cap unreacted active groups on the 2-chlorotrityl chloride resin, a solution of DCM:MeOH:DIPEA (17:2:1) was prepared and added to the previously washed and drained resin. This suspension was shaken for 30 min at room temperature. The capping solution was then filtered, and the resin was washed three times with *N*, *N*-dimethylformamide (DMF) and three times with DCM.

#### 3.1.2. Method 2. Determination of Resin Loading

CTC resin (3–4 mg) was placed in a measurement flask and 0.4 mL of DCM was added to induce swelling. After 15 min, 0.4 mL of piperidine was added and the suspension was left for additional 15 min. Next, 1.6 mL of MeOH was added and the flask was refilled with DCM. The UV absorption of the solution was read at a wavelength of 301 nm. The loading of the resin was calculated using the following equation:

(1) $$\frac{A_{301}}{7800}\;\;\;\frac{10}{m_{resin}}=\left[\frac{mmol}{g}\right]$$

The calculated value was used to determine the corresponding eq. of the reagents used for solid-phase peptide synthesis.

#### 3.1.3. Method 3. Amino Acid Coupling

All amino acids couplings were performed in a Liberty Blue CEM automatic peptide synthesizer using standard Fmoc methodology, with the appropriate Fmoc-protected amino acid (1 eq.), NMM (4.8 eq.) and DMT/NMM/TosO^-^ (1.5 eq.) as a coupling reagent. The total coupling volume was 4 mL. The resulting suspension was dispersed by nitrogen bubbling and microwaved for 5 min at 75 °C (power 30W). After coupling, three washing steps were performed with DMF 3 × 3 mL. Synthesis scale 0.1 mmol.

#### 3.1.4. Method 4. Fmoc Deprotection

Fmoc removal steps were carried out on Liberty Blue CEM in fully automatic manner. A solution of 20% piperidine in DMF (3 mL) was added to the resin. Deprotection steps were enhanced by microwave irradiation in two steps: initial deprotection (temperature 75 °C, reaction time 30 s, power 25 W) and proper deprotection (temperature 75 °C, reaction time 180 s, power 35 W). This step was combined with nitrogen mixing. The deprotection solution was drained from the reactor and three washing steps were performed with DMF 3 × 3 mL.

#### 3.1.5. Method 5. Cleavage from Resin

Linear peptides **1**-**7** were cleaved from the CTC resin using solution of 45% TFA/2.5% water/2.5% triisopropylsilane/50% DCM (2 mL/0.1 g resin) was used. The resin was filtered and washed three times with TFA 3 × 1 mL. The cleavage solution was evaporated under nitrogen gas. Precipitation of peptides was achieved by addition of cold diethyl ether. Obtained precipitate was centrifuged three times with fresh portion of cold diethyl ether, which was removed by decantation. Crude peptides were lyophilized, correct structures were confirmed by MS and reverse-phase HPLC was used to determine purity.

### 3.2. General Synthesis Procedure for Peptides 1–7

H-Leu(*N*-Me)TyrGlnLeuGluAsnTyr-OH (**1**). First C-terminal amino acids Fmoc-Tyr(tBu)-OH (114.90 mg, 0.25 mmol) was anchored to CTC resin (GM 1). Removal of Fmoc was assisted with microwave irradiation according to GM 4. After deprotection corresponding C-terminal amino acid Fmoc-Asn(Trt)-OH (179.01 mg, 0.3 mmol) was coupled according to GM 3. For each coupling DMT/NMM/TosO^-^ (206.50 mg, 0.5 mmol) and NMM (55 μL, 0.5 mmol) (GM 3) were used. Further elongation of peptide was achieved by additions of: Fmoc-Glu(OtBu)-OH (127.65 mg, 0.3 mmol); Fmoc-Leu-OH (106.02 mg, 0.3 mmol); Fmoc-Gln(Trt)-OH (183.21 mg, 0.3 mmol); Fmoc-(*N*-Me)Tyr(tBu)-OH (142.08 mg, 0.3 mmol); Fmoc-Leu-OH (106.02 mg, 0.3 mmol). After final deprotection (GM 4), the peptide was cleaved from the CTC resin (GM 5). HPLC analysis: t^R^ 2.37 min., purity of crude product >91%. LC-MS: 956.5218 [M + H]^+^, calc. 955.67).

H-LeuTyrGln(*N*-Me)LeuGluAsnTyr-OH (**2**). First C-terminal amino acids Fmoc-Tyr(tBu)-OH (114.90 mg, 0.25 mmol) was anchored to CTC resin (GM 1). Removal of Fmoc was assisted with microwave irradiation according to GM 4. After deprotection corresponding C-terminal amino acid Fmoc-Asn(Trt)-OH (179.01 mg, 0.3 mmol) was coupled according to GM 3. For each coupling DMT/NMM/TosO^-^ (206.50 mg, 0.5 mmol) and NMM (55 μL, 0.5 mmol) (GM 3) were used. Further elongation of peptide was achieved by additions of: Fmoc-Glu(OtBu)-OH (127.65 mg, 0.3 mmol); Fmoc-(*N*-Me)Leu-OH (110.22 mg, 0.3 mmol); Fmoc-Gln(Trt)-OH (183.21 mg, 0.3 mmol); Fmoc-Tyr(tBu)-OH (137.88 mg, 0.3 mmol); Fmoc-Leu-OH (106.02 mg, 0.3 mmol). After final deprotection (GM 4), the peptide was cleaved from the CTC resin (GM 5). HPLC analysis: t_R_ 2.705 min, purity of crude product >97%. LC-MS: 956.5269 [M + H]^+^, calc. 955.67).

H-Leu(*N*-Me)TyrGln(*N*-Me)LeuGluAsnTyr-OH (**3**). First C-terminal amino acids Fmoc-Tyr(tBu)-OH (114.90 mg, 0.25 mmol) was anchored to CTC resin (GM 1). Removal of Fmoc was assisted with microwave irradiation according to GM 4. After deprotection corresponding C-terminal amino acid Fmoc-Asn(Trt)-OH (179.01 mg, 0.3 mmol) was coupled according to GM 3. For each coupling DMT/NMM/TosO^-^ (206.50 mg, 0.5 mmol) and NMM (55 μL, 0.5 mmol) (GM 3) were used. Further elongation of peptide was achieved by additions of: Fmoc-Glu(OtBu)-OH (127.65 mg, 0.3 mmol); Fmoc-(*N*-Me)Leu-OH (110.22 mg, 0.3 mmol); Fmoc-Gln(Trt)-OH (183.21 mg, 0.3 mmol); Fmoc-(*N*-Me)Tyr(tBu)-OH (142.08 mg, 0.3 mmol); Fmoc-Leu-OH (106.02 mg, 0.3 mmol). After final deprotection (GM 4), the peptide was cleaved from the CTC resin (GM 5). HPLC analysis: t_R_ 3.133 min, purity of crude product >93%. LC-MS: 970.4991 [M + H]^+^, calc. 969.47).

H-ValGluAla(*N*-Me)LeuTyrLeu-OH (**4**). First C-terminal amino acids Fmoc-Leu-OH (88.35 mg, 0.25 mmol) was anchored to CTC resin (GM 1). Removal of Fmoc was assisted with microwave irradiation according to GM 4. After deprotection corresponding C-terminal amino acid Fmoc-Tyr(tBu)-OH (137.88 mg, 0.3 mmol) was coupled according to GM 3. For each coupling DMT/NMM/TosO^-^ (206.50 mg, 0.5 mmol) and NMM (55 μL, 0.5 mmol) (GM 3) were used. Further elongation of peptide was achieved by additions of: Fmoc-(*N*-Me)Leu-OH (110.22 mg, 0.3 mmol); Fmoc-Ala-OH (93.39 mg, 0.3 mmol); Fmoc-Glu(OtBu)-OH (127.65 mg, 0.3 mmol); Fmoc-Val-OH (101.82 mg, 0.3 mmol). For each coupling, DMT/NMM/TosO^-^ (206.50 mg, 0.5 mmol) and NMM (55 μL, 0.5 mmol) were used (GM 3). After final deprotection (GM 4), the peptide was cleaved from the CTC resin (GM 5). HPLC analysis: t_R_ 2.730 min, purity of crude product >96%. LC-MS: 721.4311 [M + H]^+^, calc. 720.84).

H-ValGlu(*N*-Me)AlaLeuTyrLeu-OH (**5**). First C-terminal amino acids Fmoc-Leu-OH (88.35 mg, 0.25 mmol) was anchored to CTC resin (GM 1). Removal of Fmoc was assisted with microwave irradiation according to GM 4. After deprotection corresponding C-terminal amino acid Fmoc-Tyr(tBu)-OH (137.88 mg, 0.3 mmol) was coupled according to GM 3. For each coupling DMT/NMM/TosO^-^ (206.50 mg, 0.5 mmol) and NMM (55 μL, 0.5 mmol) (GM 3) were used. Further elongation of peptide was achieved by additions of: Fmoc-Leu-OH (106.02 mg, 0.3 mmol); Fmoc-(*N*-Me)Ala-OH (97.59 mg, 0.3 mmol); Fmoc-Glu(OtBu)-OH (127.65 mg, 0.3 mmol); Fmoc-Val-OH (101.82 mg, 0.3 mmol). After final deprotection (GM 4), the peptide was cleaved from the CTC resin (GM 5). HPLC analysis: t_R_ 2.687 min, purity of crude product >98%. LC-MS: 721.3915 [M + H]^+^, calc. 720.84).

H-ValGluAlaLeu(*N*-Me)TyrLeu-OH (**6**). First C-terminal amino acids Fmoc-Leu-OH (88.35 mg, 0.25 mmol) was anchored to CTC resin (GM 1). Removal of Fmoc was assisted with microwave irradiation according to GM 4. After deprotection corresponding C-terminal amino acid Fmoc-(*N*-Me)Tyr(tBu)-OH (142.08 mg, 0.3 mmol) was coupled according to GM 3. For each coupling DMT/NMM/TosO^-^ (206.50 mg, 0.5 mmol) and NMM (55 μL, 0.5 mmol) (GM 3) were used. Further elongation of peptide was achieved by additions of: Fmoc-Leu-OH (106.02 mg, 0.3 mmol); Fmoc-Ala-OH (93.39 mg, 0.3 mmol); Fmoc-Glu(OtBu)-OH (127.65 mg, 0.3 mmol); Fmoc-Val-OH (101.82 mg, 0.3 mmol). After final deprotection (GM 4), the peptide was cleaved from the CTC resin (GM 5). HPLC analysis: t_R_ 2.730 min, purity of crude product >96%. LC-MS: 721.4386 [M + H]^+^, calc. 720.84).

H-ValGlu(*N*-Me)AlaLeu(*N*-Me)TyrLeu-OH (**7**). First C-terminal amino acids Fmoc-Leu-OH (88.35 mg, 0.25 mmol) was anchored to CTC resin (GM 1). Removal of Fmoc was assisted with microwave irradiation according to GM 4. After deprotection corresponding C-terminal amino acid Fmoc-(*N*-Me)Tyr(tBu)-OH (142.08 mg, 0.3 mmol) was coupled according to GM 3. For each coupling DMT/NMM/TosO^-^ (206.50 mg, 0.5 mmol) and NMM (55 μL, 0.5 mmol) (GM 3) were used. Further elongation of peptide was achieved by additions of: Fmoc-Leu-OH (106.02 mg, 0.3 mmol); Fmoc-(*N*-Me)Ala-OH (97.59 mg, 0.3 mmol); Fmoc-Glu(OtBu)-OH (127.65 mg, 0.3 mmol); Fmoc-Val-OH (101.82 mg, 0.3 mmol). After final deprotection (GM 4), the peptide was cleaved from the CTC resin (GM 5). HPLC analysis: t_R_ 2.720 min, purity of crude product >98%. LC-MS: 734.8776 [M + H]^+^, calc. 734.84).

### 3.3. General Methods for Assays with Congo Red and Thioflavin T to Determine Aggregation Propensity

#### 3.3.1. Spectroscopic Measurements

All UV-Vis spectra to determine the progression of aggregation by the synthesized peptides were obtained using a Hitachi spectrophotometer (Hitachi, Tokyo, Japan), with readings in the range of 400–800 nm.

Each of the synthesized peptides **1**–**3** (final concentration 2.88 mM), mixtures of hot-spot A with each peptide **1**–**3** and hot-spot B (1.44 mM) with each peptide **4**–**7** (1.44 mM), as well as mixtures of insulin (1.44 mM) with one of the peptides **1**–**7** (1.44 mM), were kept in phosphate buffer solution at pH 7.2 (1 mL) for 7 days at a temperature of 37.2 °C. After 7 days, 1 mL of a solution of CR (45 µM in phosphate buffer, pH 7.2) was added to each incubated sample. Incubation was continued for another 4 days. During this period, spectroscopic measurements of all incubated samples were performed in the range of 400–800 nm. A mixture of CR solution (45 µM in phosphate buffer, pH 7.2, 1 mL) and phosphate buffer (pH 7.2, 1 mL) was used as a reference sample. The UV spectra obtained in the presence of Congo Red dye (CR) on the first, second, third, and fourth days of incubation for peptides 1–7, as well as for the mixtures of hot-spots A or B with peptides **1**–**3** and **4**–**7**, respectively, and for the mixtures of insulin with peptides **1**–**7** are presented in the Supporting Material. UV spectra of **1**–**7** peptides—Figures S15–S21; complexes 1:1 of **1**–**3** peptides with hot-spot A—Figures S22–S24; complexes 1:1 of **4**–**7** peptides with hot-spot B—Figures S25–S28; complexes 2:1 of **1**–**3** peptides with hot-spot A—Figures S29–S31; complexes 2:1 of **4**–**7** peptides with hot-spot B—Figures S32–S35; complexes 1:1 of **1**–**7** peptides with insulin—Figures S36–S42.

#### 3.3.2. Fluorescence Measurements

All fluorescence spectra to determine the progression of aggregation by the synthesized peptides were obtained using a FLUOROMAX-4 system from Horiba Scientific (Edison, NJ, USA) with readings in the range of 470–600 nm. Excitation wavelength 440 nm.

Each of the synthesized peptides **1**–**7** (final concentration 0.66 mM), as well as the mixtures of hot-spot A with each peptide **1**–**3** (0.33 mM) and hot-spot B (0.33 mM) with each peptide **4**–**7** (0.33 mM) and the mixtures of insulin (0.33 mM) with one of the peptides **1**–**7** (0.33 mM), were incubated in phosphate buffer solution, pH 6.0 (2 mL), for 7 days at a temperature of 37.2 °C. After 7 days, 2 mL of a solution of Thioflavin T (57 mM in phosphate buffer, pH 6.0) was added to the incubated samples. Incubation was continued for another 4 days. During this period, spectroscopic measurements of all the incubated samples were performed in the range of 470–600 nm, excitation wavelength 440 nm. A mixture of Thioflavin T solution (57 mM in phosphate buffer, pH 6.0, 2 mL) and phosphate buffer (pH 7.2, 2 mL) was used as a reference sample. Fluorescence intensity spectra obtained on the first, second, third, and fourth days of incubation for peptides **1**–**7**, as well as for the mixtures of hot-spots A and B with peptides **1**–**3** and **4**–**7**, respectively, and for the mixtures of insulin with peptides **1**–**7** in the presence of ThT, are presented in the Supporting Material. Fluorescence intensity spectra of **1**–**7** peptides—Figures S43–S49; complexes 1:1 of **1**–**3** peptides with hot-spot A—Figures S50–S52; complexes 1:1 of **4**–**7** peptides with hot-spot B—Figures S53–S56; complexes 2:1 of **1**–**3** peptides with hot-spot A—Figures S57–S59; complexes 2:1 of **4**–**7** peptides with hot-spot B—Figures S60–S63; complexes 1:1 of **1**–**7** peptides with insulin—Figures S64–S70.

### 3.4. Microscopic Measurements after Incubation with Congo Red

Microscope probes were obtained after incubation of the peptides with CR dye. The samples were centrifuged twice and washed with distilled water. Next, all the peptides were placed with a small amount of distilled water on microscopic slides and protected with a covering glass. All microscopic measurements were performed with and without a polarized filter using a Delta Optical Genetic Pro microscope (Warsaw, Poland).

## 4. Conclusions

These studies aimed to design and synthesize fragments of the native insulin hormone and their analogs containing *N*-methylated amino acid residues, which were then evaluated for their ability to inhibit insulin aggregation. It was found that the incorporation of *N*-methylated amino acid residues into the peptide chains of fragments A13–A19 and B12–B17 (hot-spots A and B) produces inhibitors of insulin aggregation (see Table 1). Peptides **1**–**7** effectively inhibit insulin aggregation. However, although the propensity of peptides **1**–**7** to aggregate was significantly lower in comparison to the native fragments, inhibition was less effective in the case of aggregation by both hot-spots A and B in the presence of peptides **1**–**7**, in comparison to the native hormone. This may be explained by the greater stability of the spatial structure of insulin in comparison to the short fragments A13–A19 and B12–B17, which facilitates its interaction with potential inhibitors. An important finding was that peptides **1**–**7**, which inhibited the aggregation of native insulin, themselves can aggregate. In most cases, this susceptibility is significantly lower than that of the native hot-spots A and B. Some *N*-methylated analogs, on the other hand, showed increased ability to aggregate (peptides **1**–**3**, ThT assay).

Designing inhibitors of insulin aggregation could prevent the destruction of β-cells in the pancreas. Insoluble protein deposit formation proceeds by a common mechanism in conformational diseases. It therefore seems possible that the development of an effective method for inhibiting the aggregation of a protein/polypeptide associated with one disease could be adapted to design inhibitors of aggregation by other amyloidogenic proteins. The new inhibitors of insulin aggregation proposed in this work may in addition be characterized by increased resistance to proteolytic enzymes, due to the presence of unnatural amino acids in the peptide chain. Based on three independent tests, six out of the seven tested peptides (except for peptide **2**) meet the criteria for potential inhibitors of aggregation (at least two tests must confirm the absence of aggregation). Peptides **1** and **3**–**7** may be suitable for use as insulin stabilizing systems, increasing the shelf life of medication for diabetics.

## Figures and Tables

**Table 1:** Summary of results: inhibition of insulin aggregation by peptides **1**–**7**.

Insulin Complex with Peptides 1–7	CR Assay	ThT Assay	Microscopic Examination
Complex (1:1) H-Leu(*N*-Me)TyrGlnLeuGluAsnTyr-OH (**1**) with Insulin	-	-	-
Complex (1:1) H-LeuTyrGln(*N*-Me)LeuGluAsnTyr-OH (**2**) with Insulin	+/−	+/−	-
Complex (1:1) H-Leu(*N*-Me)TyrGln(*N*-Me)LeuGluAsnTyr-OH (**3**) with Insulin	-	+/−	-
Complex (1:1) H-ValGluAla(*N*-Me)LeuTyrLeu-OH (**4**) with Insulin	-	-	-
Complex (1:1) H-ValGlu(*N*-Me)AlaLeuTyrLeu-OH (**5**) with Insulin	-	-	-
Complex (1:1) H-ValGluAlaLeu(*N*-Me)TyrLeu-OH (**6**) with Insulin	-	-	-
Complex (1:1) H-ValGlu(*N*-Me)AlaLeu(*N*-Me)TyrLeu-OH (**7**) with Insulin	+/−	-	-

## Supplementary Materials

The following are available online, the HPLC of crude **1**–**7** peptides (Figures S1, 3, 5, 7, 9, 11, 13); MS spectra of **1**–**7** peptides (Figures S2, 4, 6, 8, 10, 12, 14); UV spectra of **1**–**7** peptides and their complexes with hot-spots A and B (Figures S15–35 and with insulin (Figures S36–42); fluorescence intensity spectra of **1**–**7** peptides and their complexes with hot-spots A and B (Figures S43–63) and with insulin (Figures S64–70).
