# Supplementary material for: Insulin Hot-Spot Analogs Formed with N-Methylated Amino Acid Residues Inhibit Aggregation of Native Hormone
Source: Molecules. 2019 Oct 15;24(20):3706. doi: 10.3390/molecules24203706 (PMC6832904; doi:10.3390/molecules24203706)
Supplement: Supplementary file 1 [file molecules-24-03706-s001.pdf]

Hot spot analogues formed with *N*-methylated amino acid residues inhibit insulin aggregation

Monika Swiontek[1], Joanna Wasko[1], Justyna Fraczyk[1], Krystian Galecki[2], Zbigniew J. Kaminski[1], and Beata Kolesinska[1,*]

[1)] Institute of Organic Chemistry, Faculty of Chemistry, Lodz University of Technology, Zeromskiego 116, 90-924 Lodz
[2)] Institute of General Food Chemistry, Faculty of Biotechnology & Food Sciences, Lodz University of Technology, Stefanowskiego 4/10, 90-924 Lodz, Poland

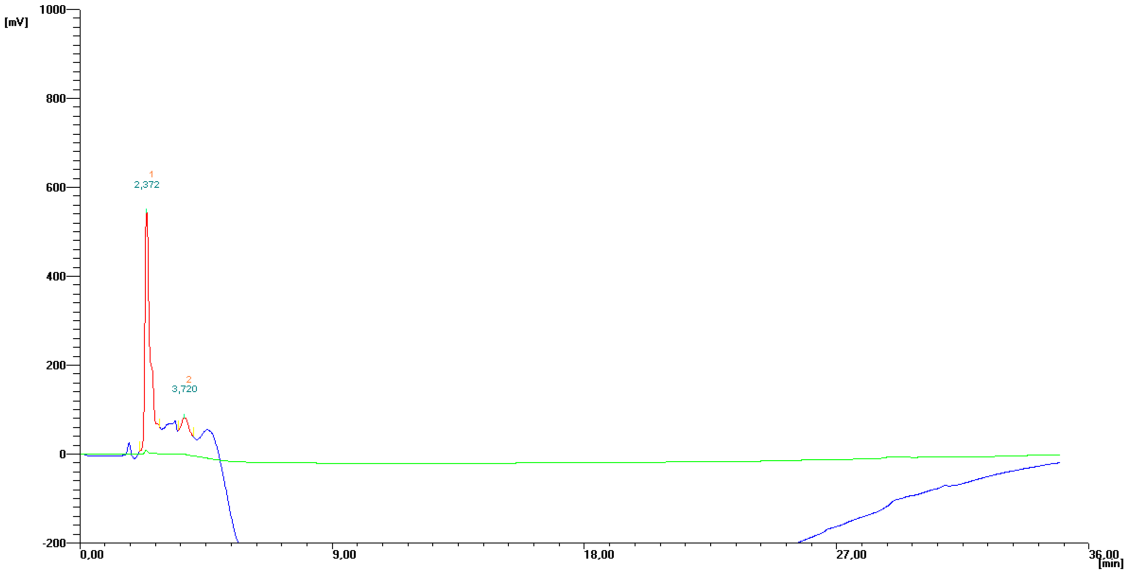

Fig. S1. Chromatograph HPLC of H_2N-Leu(*N*-Me)TyrGlnLeuGluAsnTyr-COOH (**1**).

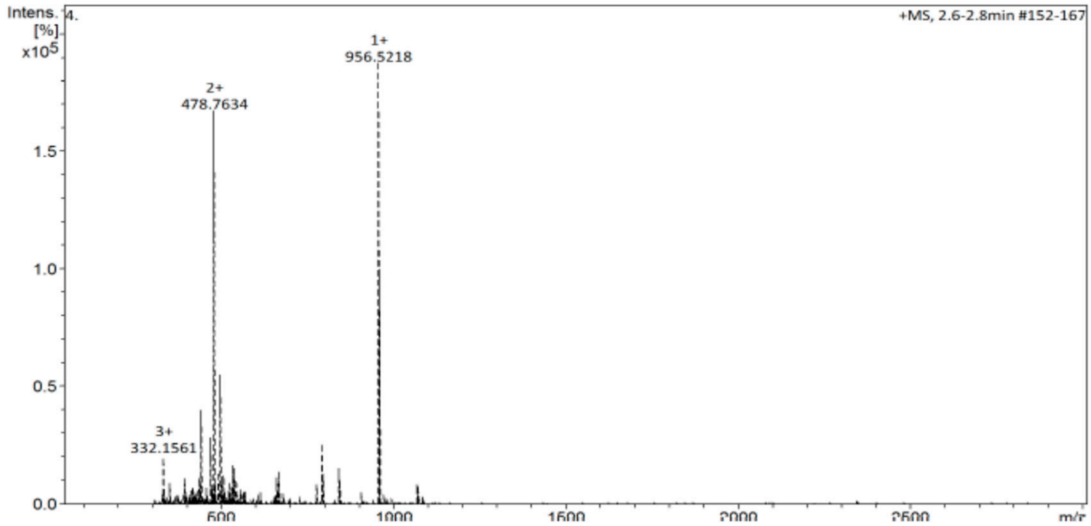

Fig. S2. MS spectra of H_2N-Leu(*N*-Me)TyrGlnLeuGluAsnTyr-COOH (**1**).

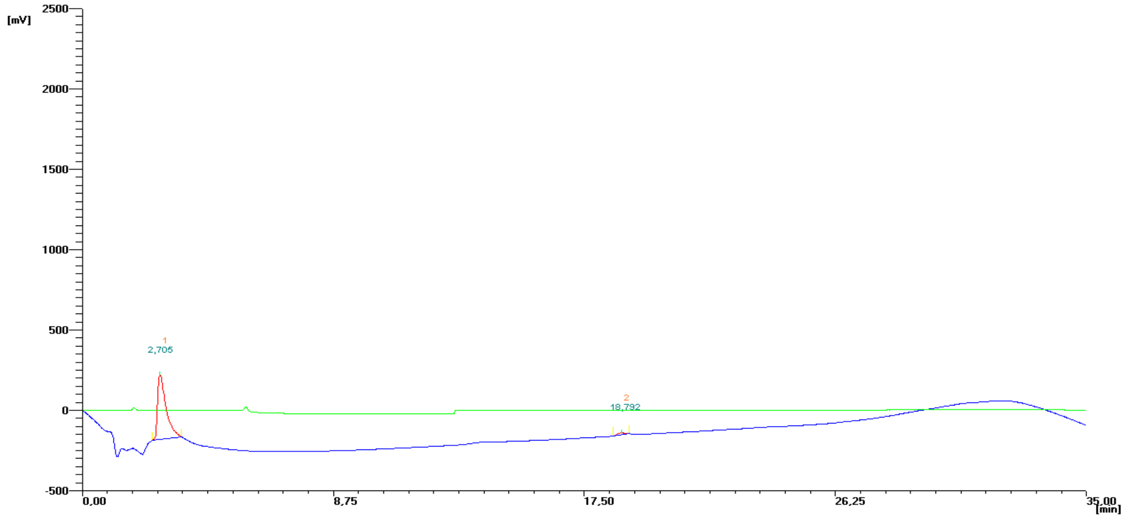

Fig. S3. Chromatograph HPLC of H_2N-LeuTyrGln(*N*-Me)LeuGluAsnTyr-COOH (**2**).

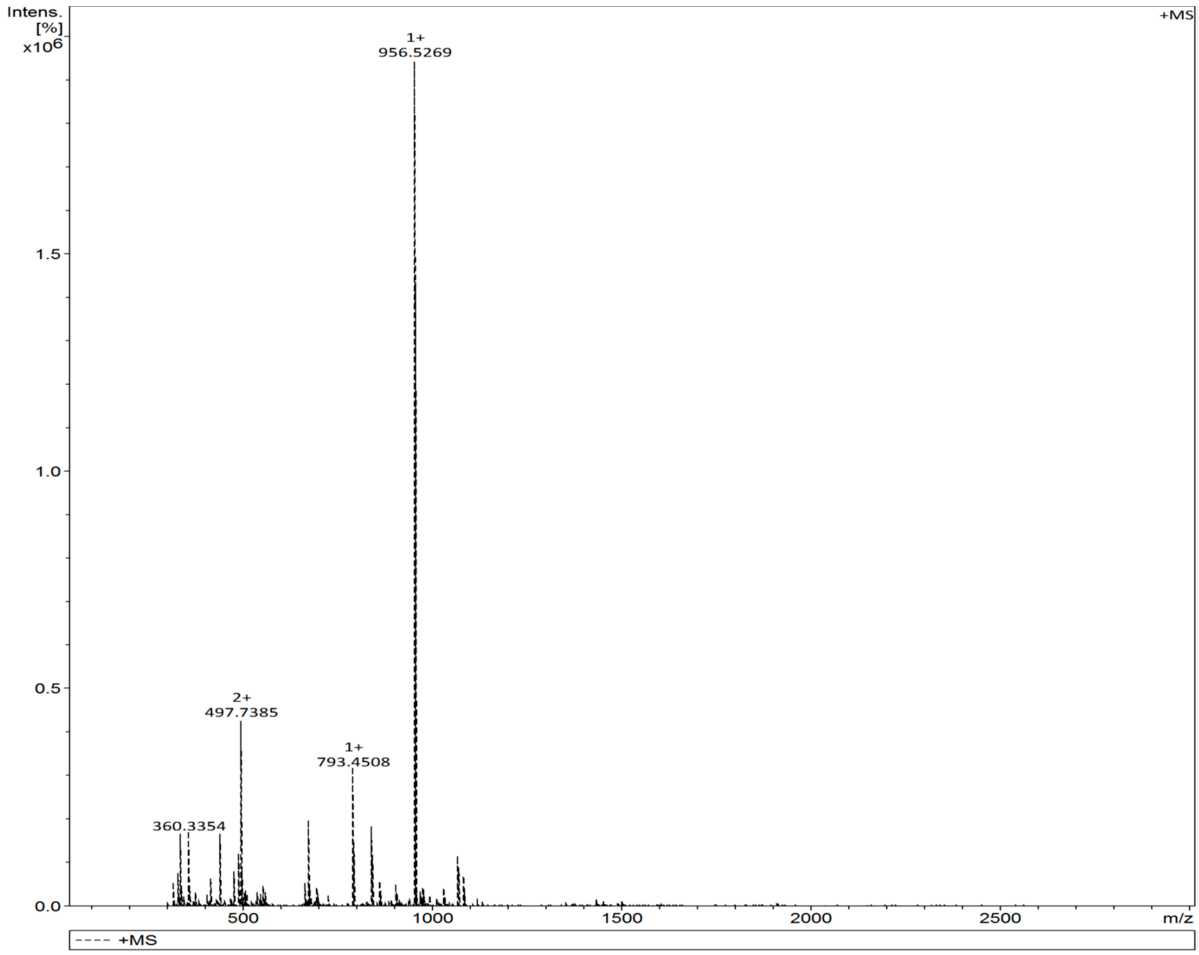

Fig. S4. MS spectra of H_2N-LeuTyrGln(*N*-Me)LeuGluAsnTyr-COOH (**2**).

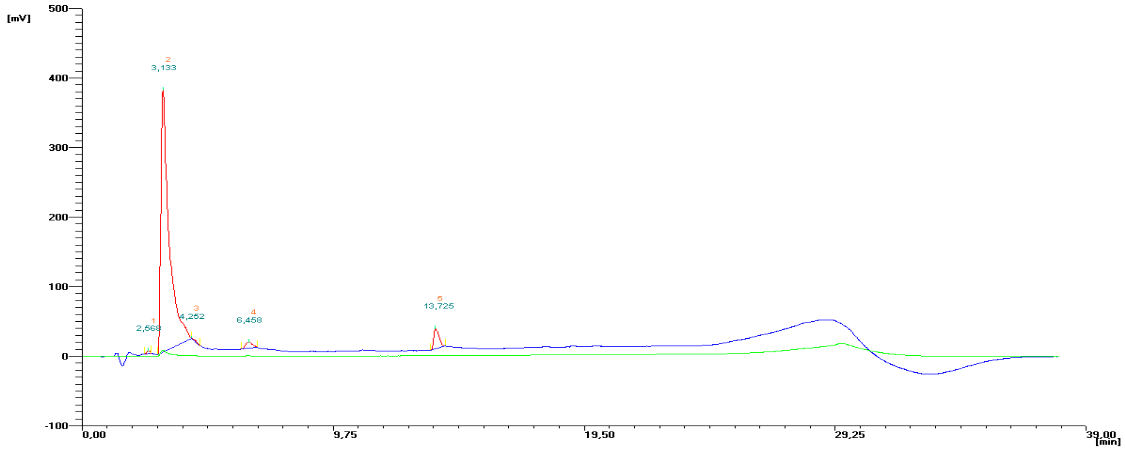

Fig. S5. Chromatograph HPLC of H_2N-Leu(*N*-Me)TyrGln(*N*-Me)LeuGluAsnTyr-COOH (**3**).

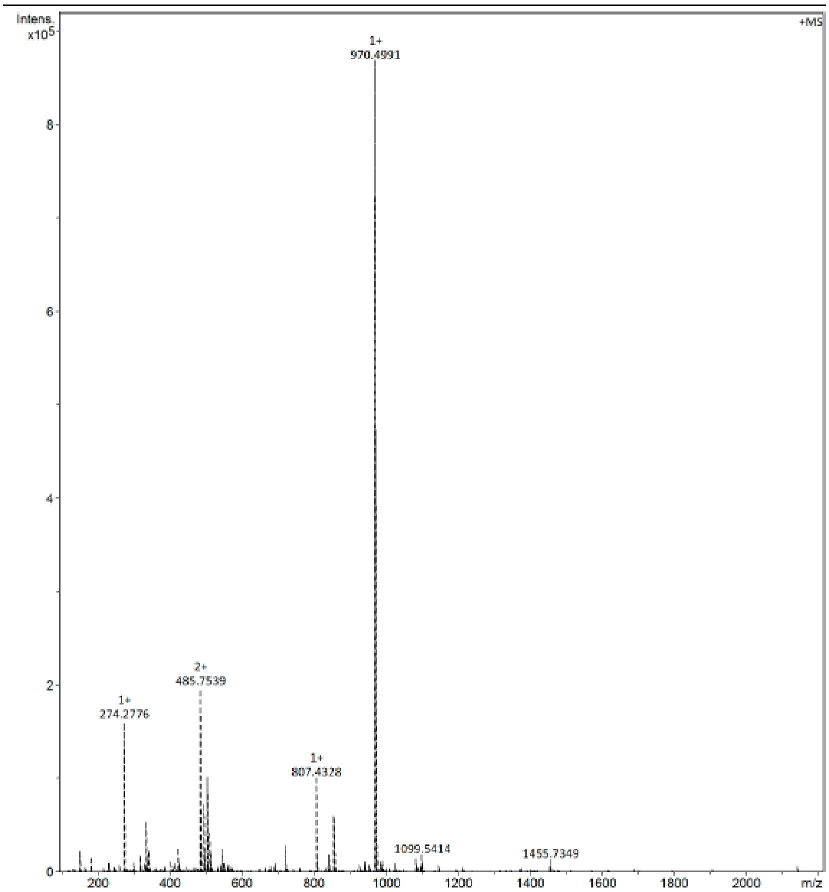

Fig. S6. MS spectra of H_2N-Leu(*N*-Me)TyrGln(*N*-Me)LeuGluAsnTyr-COOH (**3**).

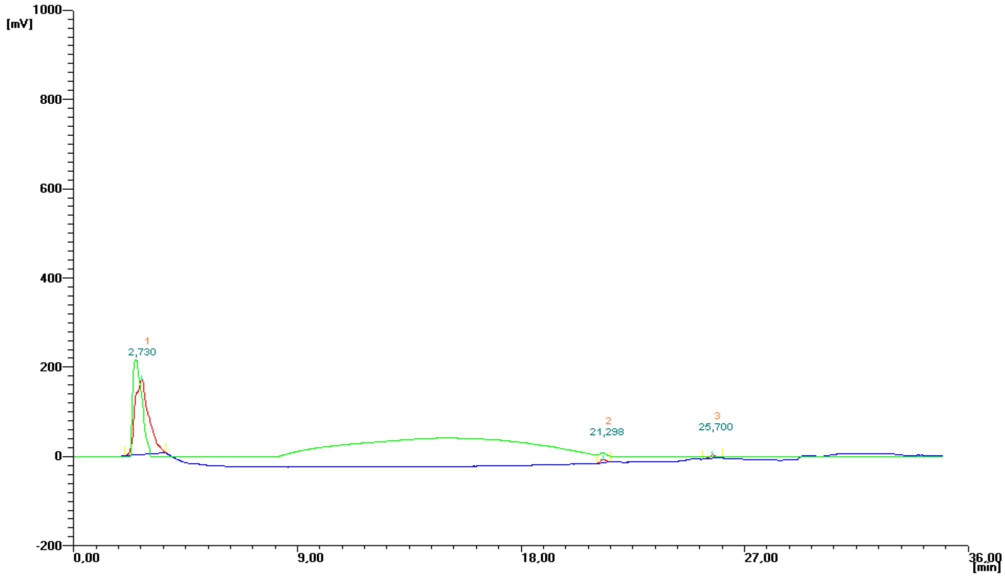

Fig. S7. Chromatograph HPLC of H_2N-ValGluAla(*N*-Me)LeuTyrLeu-COOH (**4**).

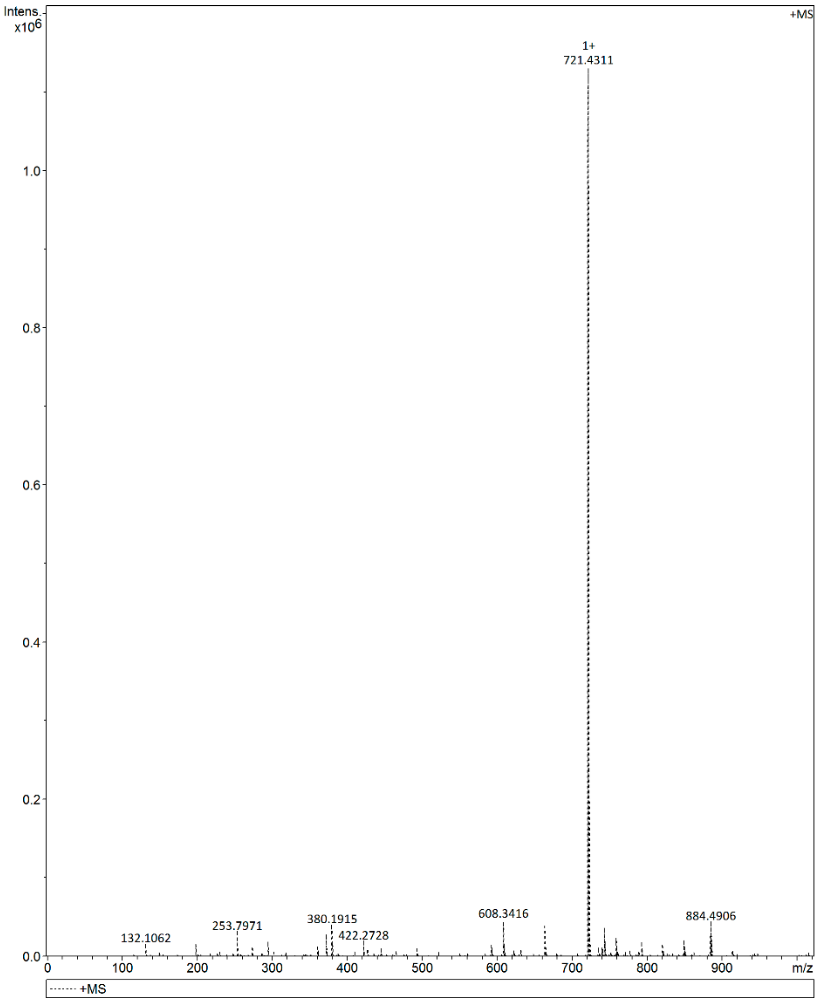

Fig. S8. MS spectra of H_2N-ValGluAla(*N*-Me)LeuTyrLeu-COOH (**4**).

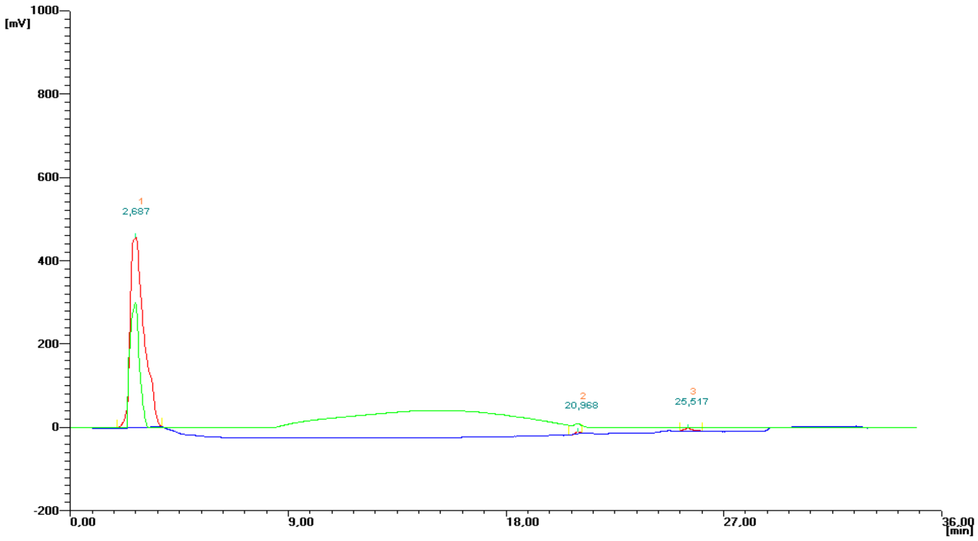

Fig. S9. Chromatograph HPLC of H_2N-ValGlu(*N*-Me)AlaLeuTyrLeu-COOH (**5**).

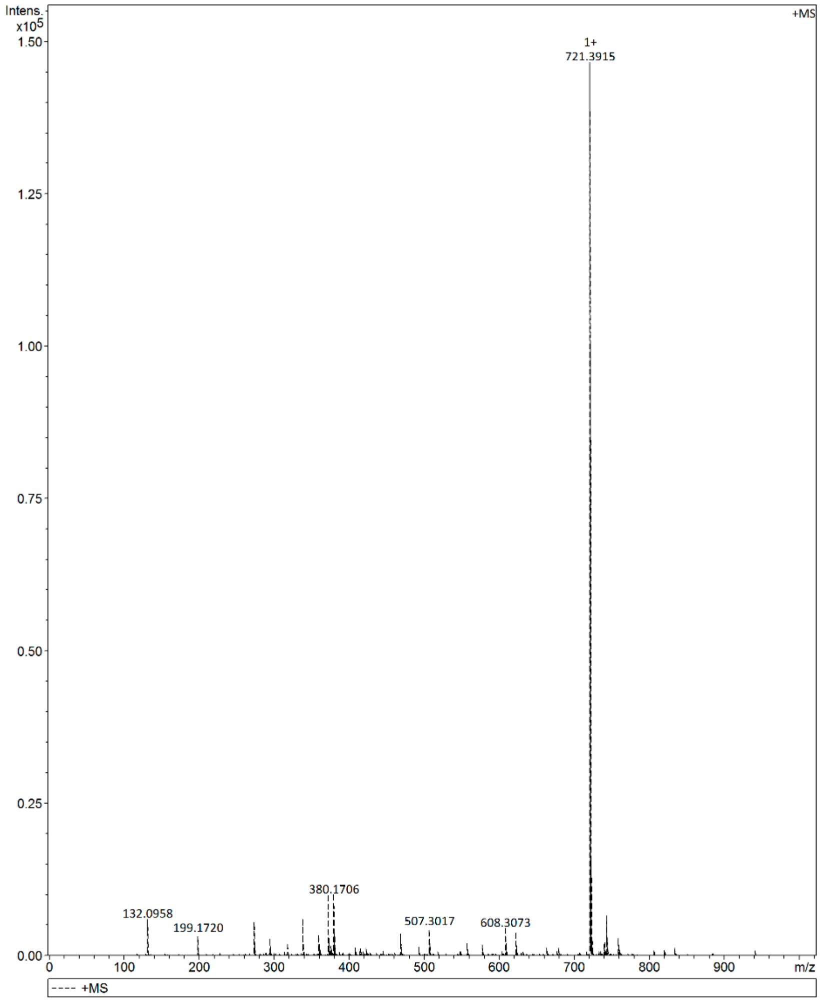

Fig. S10. MS spectra of H_2N-ValGlu(*N*-Me)AlaLeuTyrLeu-COOH (**5**).

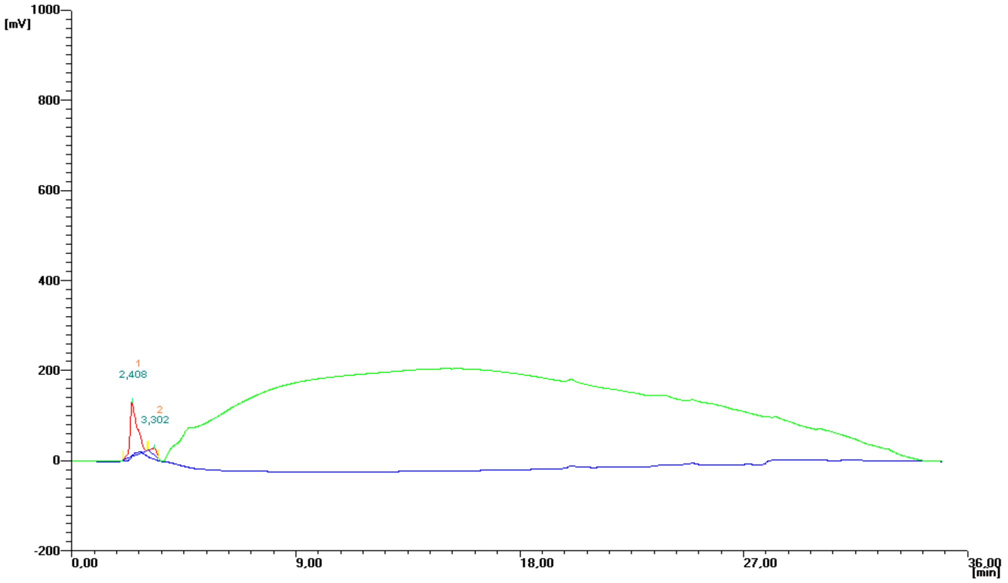

Fig. S11. Chromatograph HPLC of H_2N-ValGluAlaLeu(*N*-Me)TyrLeu-COOH (**6**).

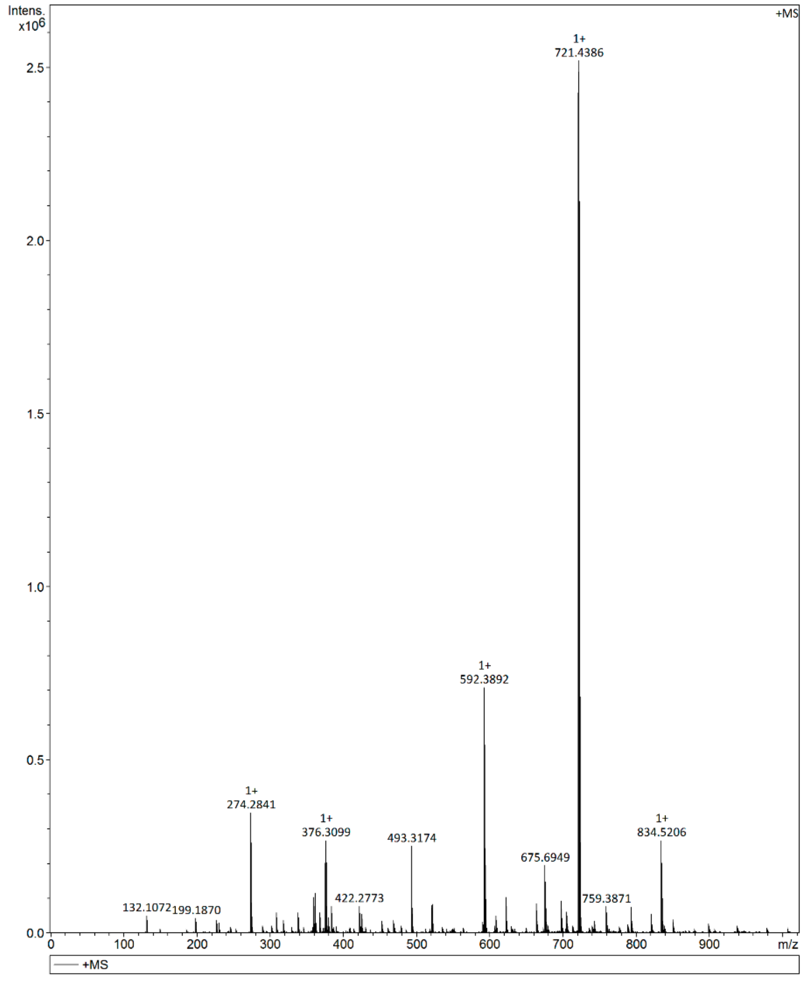

Fig. S12. MS spectra of H_2N-ValGluAlaLeu(*N*-Me)TyrLeu-COOH (**6**).

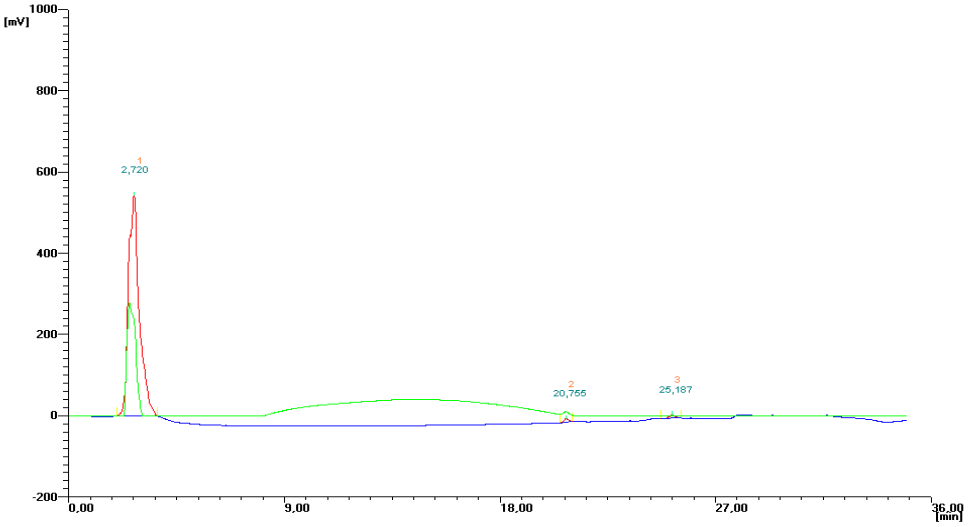

Fig. S13. Chromatograph HPLC of H_2N-ValGlu(*N*-Me)AlaLeu(*N*-Me)TyrLeu-COOH (**7**).

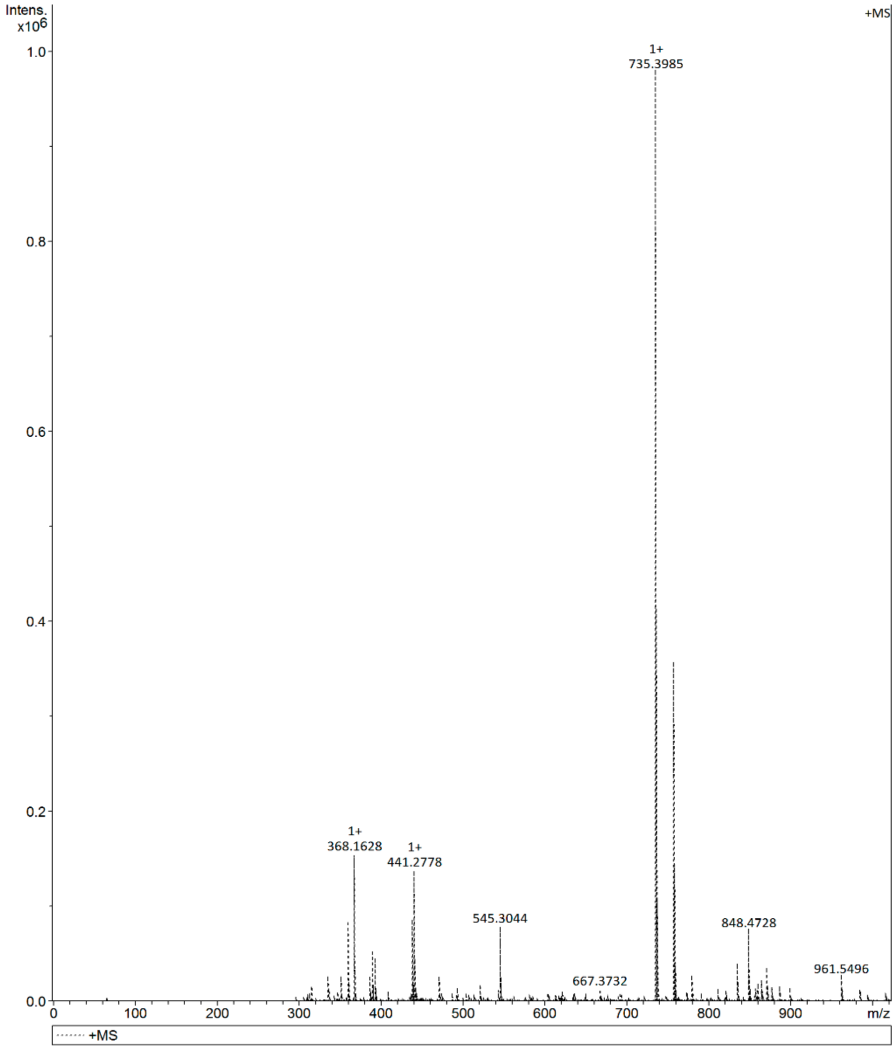

Fig. S14. MS spectra of H_2N-ValGlu(*N*-Me)AlaLeu(*N*-Me)TyrLeu-COOH (**7**).

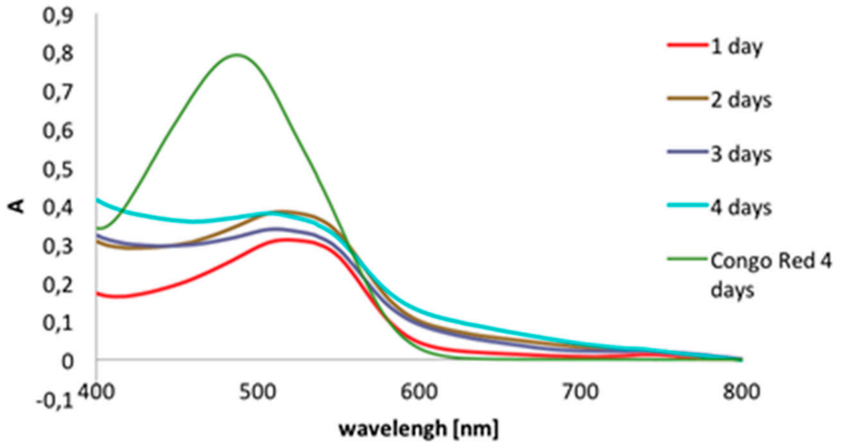

Fig. S15. UV spectra of H_2N-Leu(*N*-Me)TyrGlnLeuGluAsnTyr-COOH (**1**).

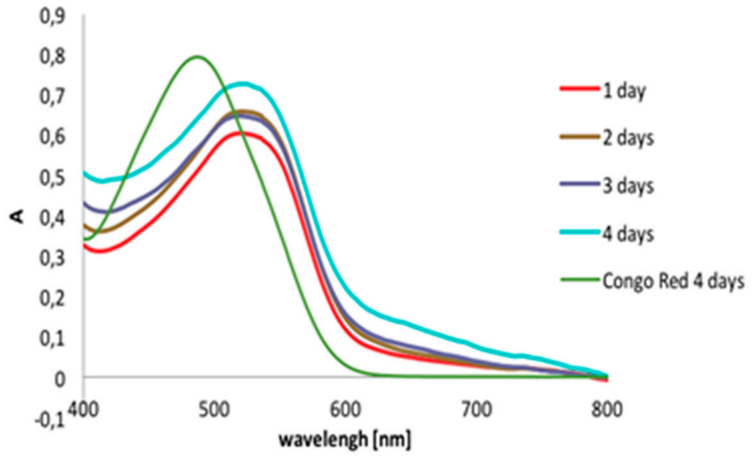

Fig. S16. UV spectra of H_2N-LeuTyrGln(*N*-Me)LeuGluAsnTyr-COOH (**2**).

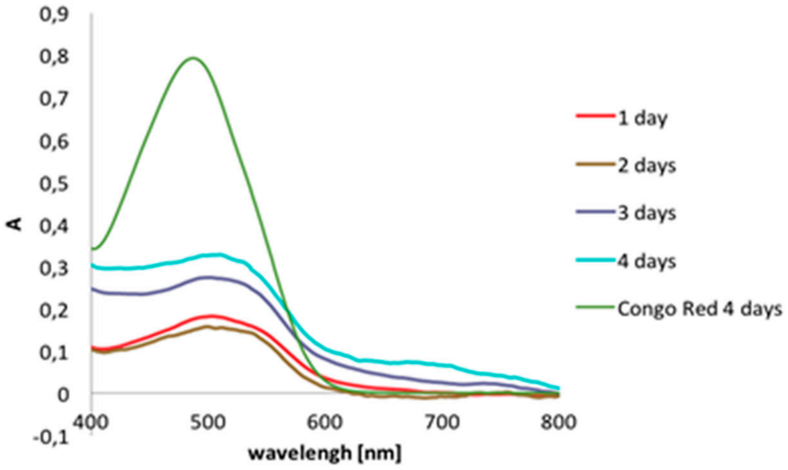

Fig. S17. UV spectra of H_2N-Leu(*N*-Me)TyrGln(*N*-Me)LeuGluAsnTyr-COOH (**3**).

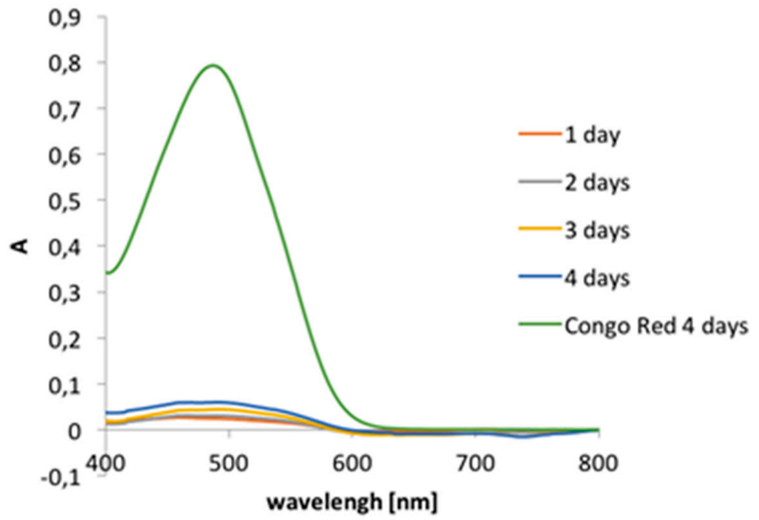

Fig. S18. UV spectra of H_2N-ValGluAla(*N*-Me)LeuTyrLeu-COOH (**4**).

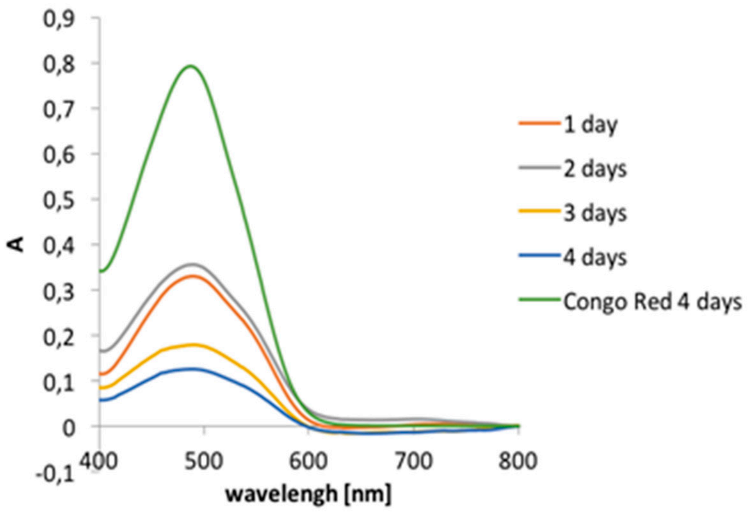

Fig. S19. UV spectra of H_2N-ValGlu(*N*-Me)AlaLeuTyrLeu-COOH (**5**).

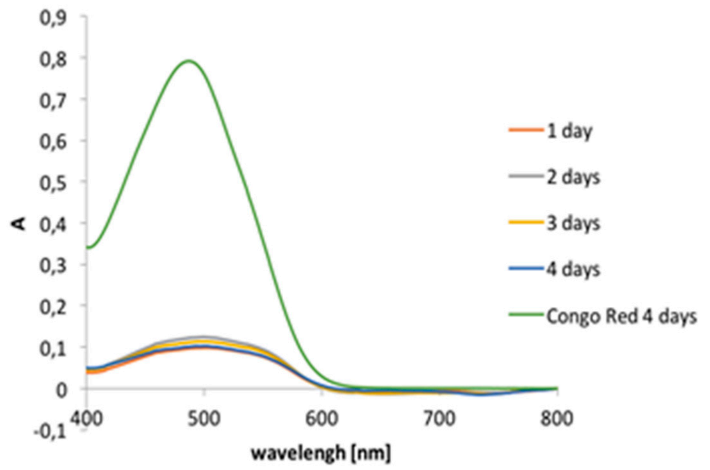

Fig. S20. UV spectra of H_2N-ValGluAlaLeu(*N*-Me)TyrLeu-COOH (**6**).

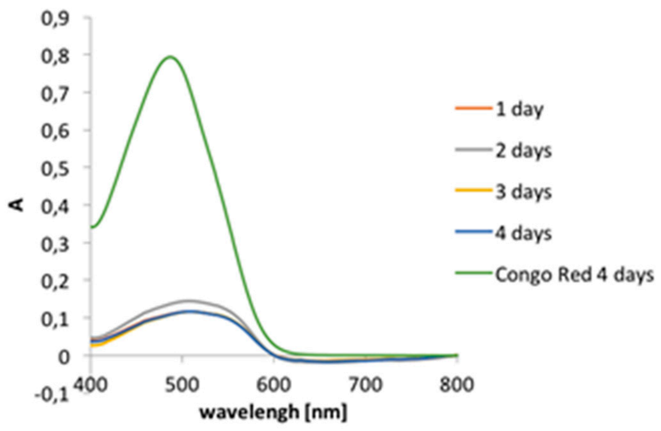

Fig. S21. UV spectra of H_2N-ValGlu(*N*-Me)AlaLeu(*N*-Me)TyrLeu-COOH (**7**).

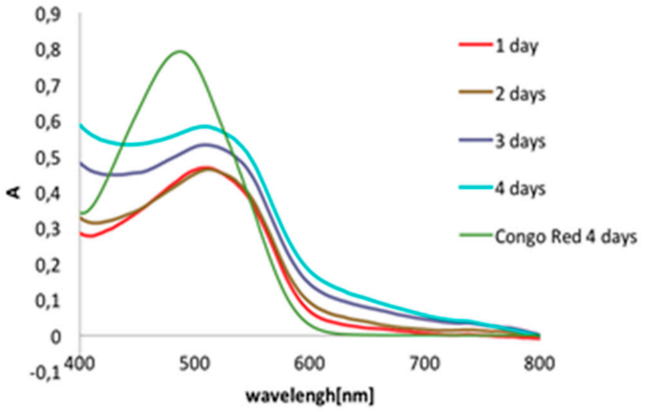

Fig. S22. UV spectra of mixture of H_2N-Leu(*N*-Me)TyrGlnLeuGluAsnTyr-COOH (**1**) with hot spot A (1:1).

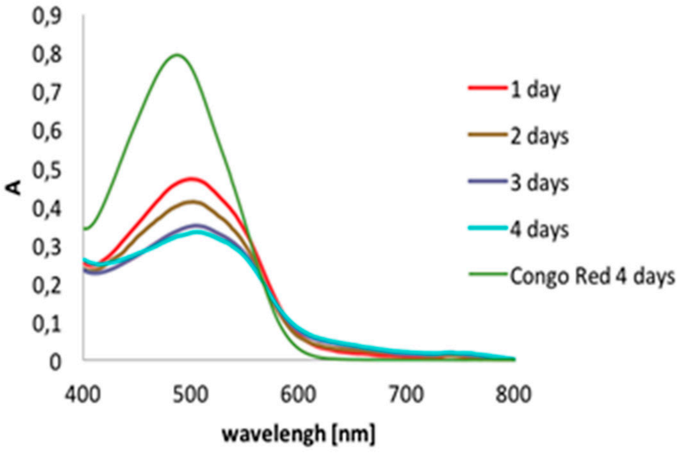

Fig. S23. UV spectra of mixture of H_2N-LeuTyrGln(*N*-Me)LeuGluAsnTyr-COOH (**2**) with hot spot A (1:1).

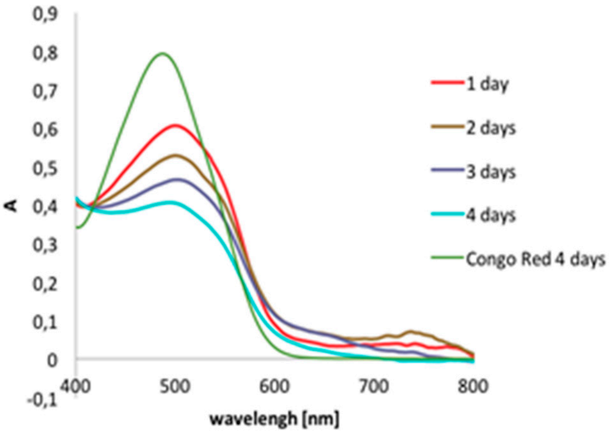

Fig. S24. UV spectra of mixture of H_2N-Leu(*N*-Me)TyrGln(*N*-Me)LeuGluAsnTyr-COOH (**3**) with hot spot A (1:1).

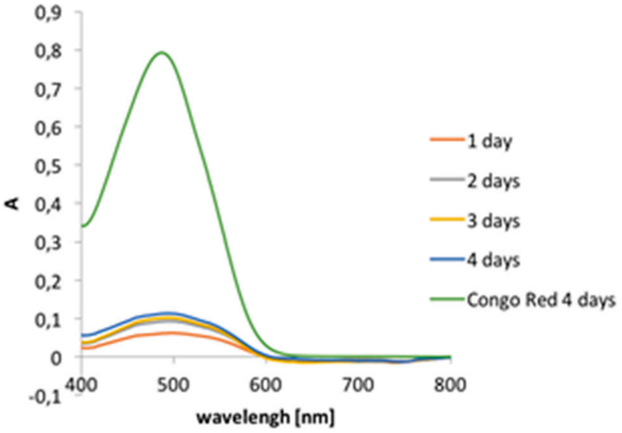

Fig. S25. UV spectra of mixture of H_2N-ValGluAla(*N*-Me)LeuTyrLeu-COOH (**4**) with hot spot B (1:1).

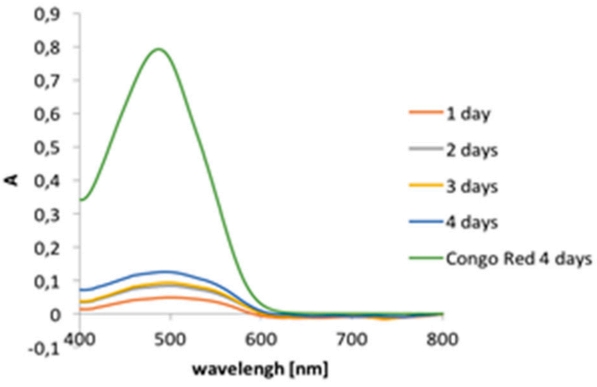

Fig. S26. UV spectra of mixture of H_2N-ValGlu(*N*-Me)AlaLeuTyrLeu-COOH (**5**) with hot spot B (1:1).

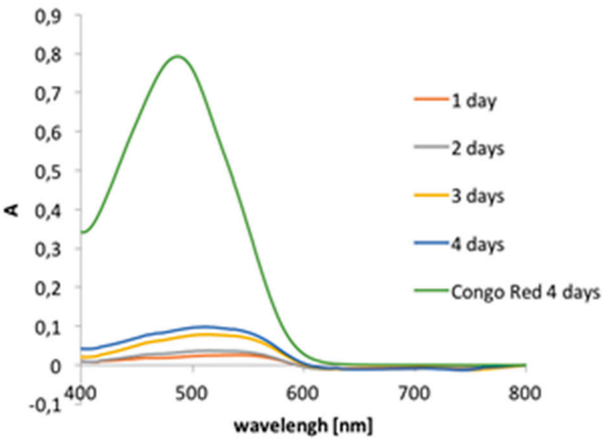

Fig. S27. UV spectra of mixture of H_2N-ValGluAlaLeu(*N*-Me)TyrLeu-COOH (**6**) with hot spot B (1:1).

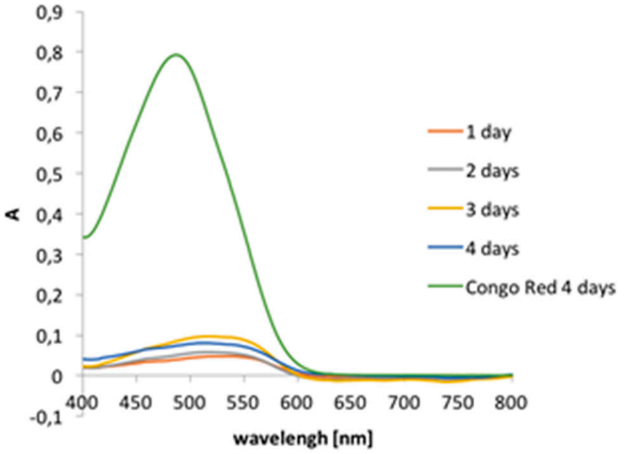

Fig. S28. UV spectra of mixture of H_2N-ValGlu(*N*-Me)AlaLeu(*N*-Me)TyrLeu-COOH (**7**) with hot spot B (1:1).

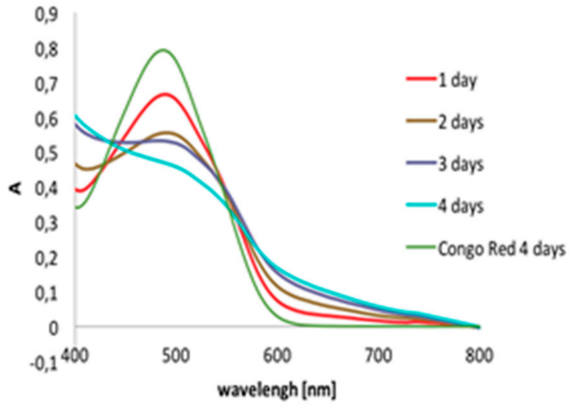

Fig. S29. UV spectra of mixture of H_2N-Leu(*N*-Me)TyrGlnLeuGluAsnTyr-COOH (**1**) with hot spot A (2:1).

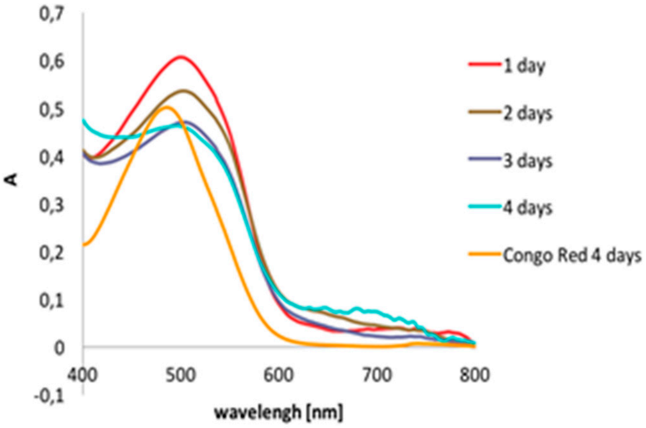

Fig. S30. UV spectra of mixture of H_2N-LeuTyrGln(*N*-Me)LeuGluAsnTyr-COOH (**2**) with hot spot A (2:1).

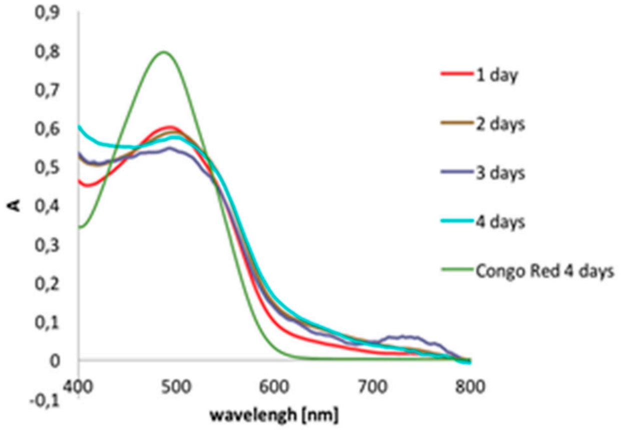

Fig. S31. UV spectra of mixture of H_2N-Leu(*N*-Me)TyrGln(*N*-Me)LeuGluAsnTyr-COOH (**3**) with hot spot A (2:1).

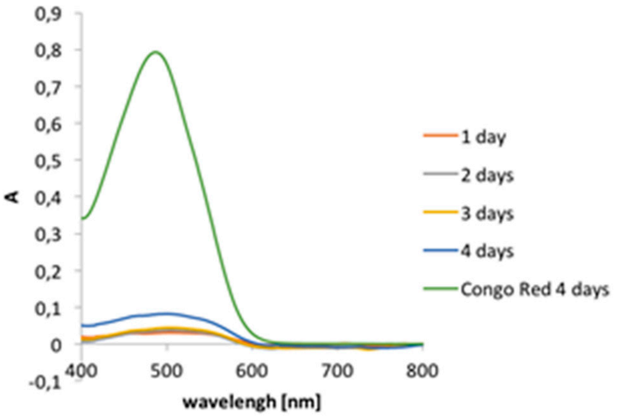

Fig. S32. UV spectra of mixture of H_2N-ValGluAla(*N*-Me)LeuTyrLeu-COOH (**4**) with hot spot B (2:1).

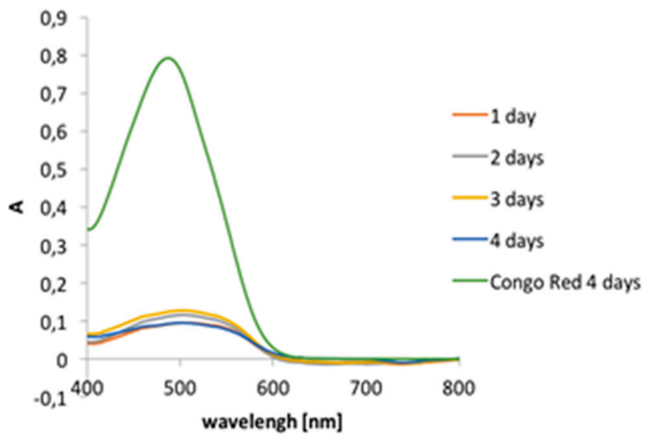

Fig. S33. UV spectra of mixture of H_2N-ValGlu(*N*-Me)AlaLeuTyrLeu-COOH (**5**) with hot spot A (2:1).

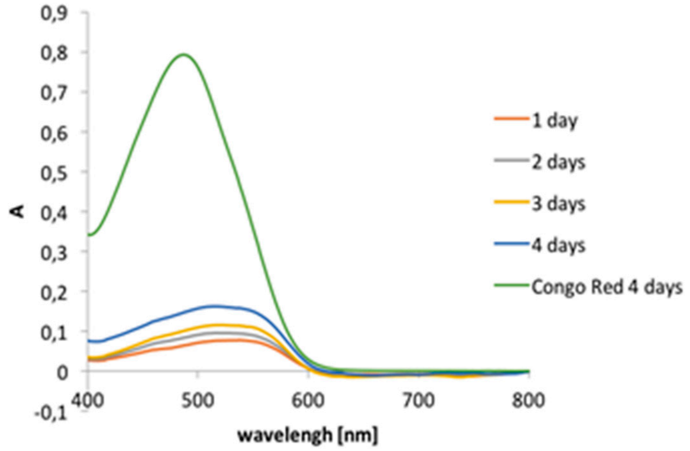

Fig. S34. UV spectra of mixture of H_2N-ValGluAlaLeu(*N*-Me)TyrLeu-COOH (**6**) with hot spot A (2:1).

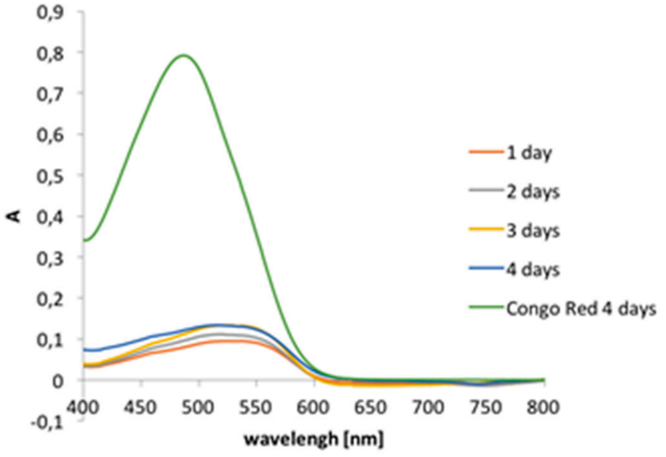

Fig. S35. UV spectra of mixture of H_2N-ValGlu(*N*-Me)AlaLeu(*N*-Me)TyrLeu-COOH (**7**) with hot spot A (2:1).

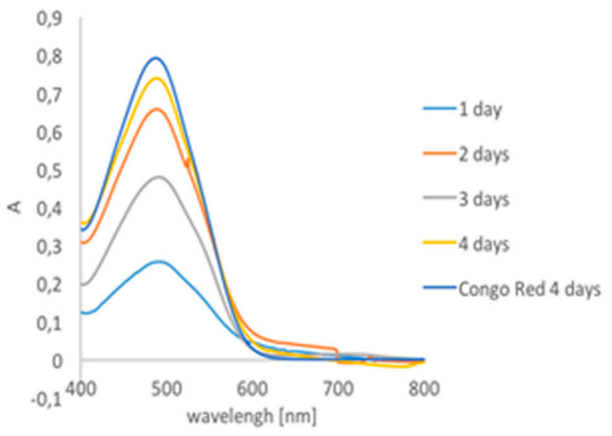

Fig. S36. UV spectra of mixture of H_2N-Leu(*N*-Me)TyrGlnLeuGluAsnTyr-COOH (**1**) with insulin (1:1).

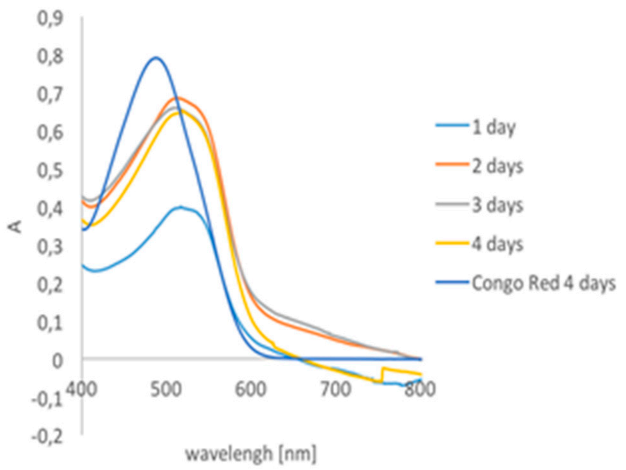

Fig. S37. UV spectra of mixture of H_2N-LeuTyrGln(*N*-Me)LeuGluAsnTyr-COOH (**2**) with insulin (1:1).

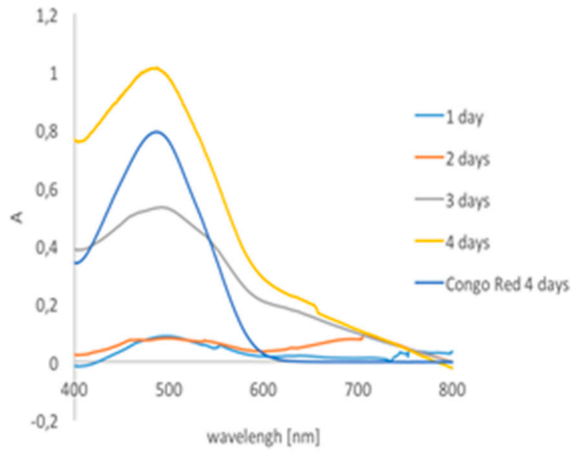

Fig. S38. UV spectra of mixture of H_2N-Leu(*N*-Me)TyrGln(*N*-Me)LeuGluAsnTyr-COOH (**3**) with insulin (1:1).

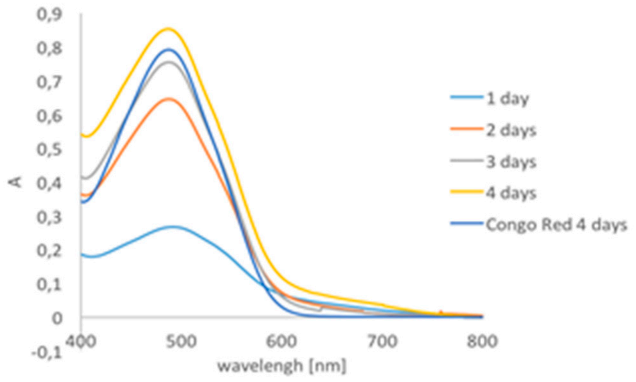

Fig. S39. UV spectra of mixture of H_2N-ValGluAla(*N*-Me)LeuTyrLeu-COOH (**4**) with insulin (1:1).

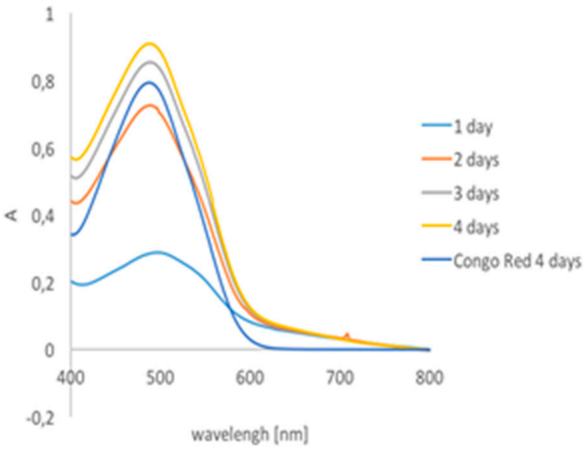

Fig. S40. UV spectra of mixture of H_2N-ValGlu(*N*-Me)AlaLeuTyrLeu-COOH (**5**) with insulin (1:1).

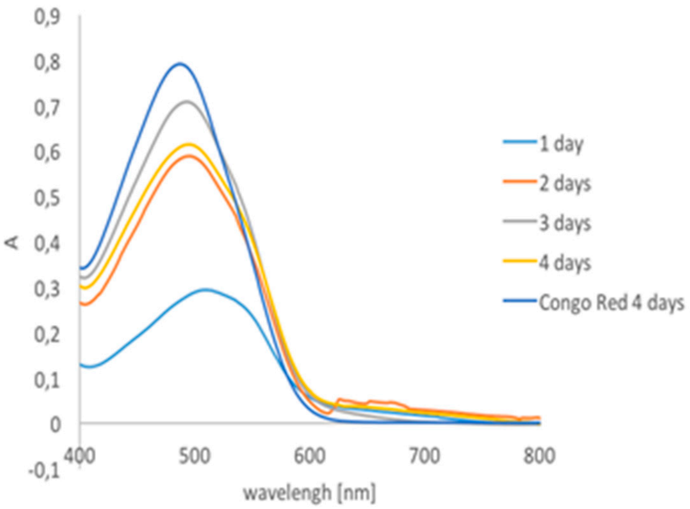

Fig. S41. UV spectra of mixture of H_2N-ValGluAlaLeu(*N*-Me)TyrLeu-COOH (**6**) with insulin (1:1).

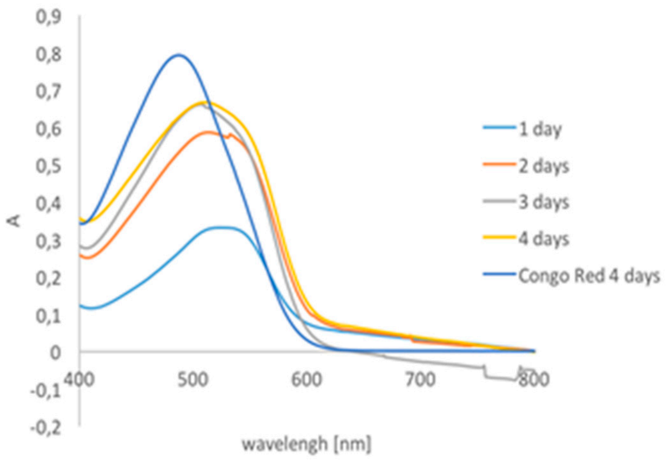

Fig. S42. UV spectra of mixture of H_2N-ValGlu(*N*-Me)AlaLeu(*N*-Me)TyrLeu-COOH (**7**) with insulin (1:1).

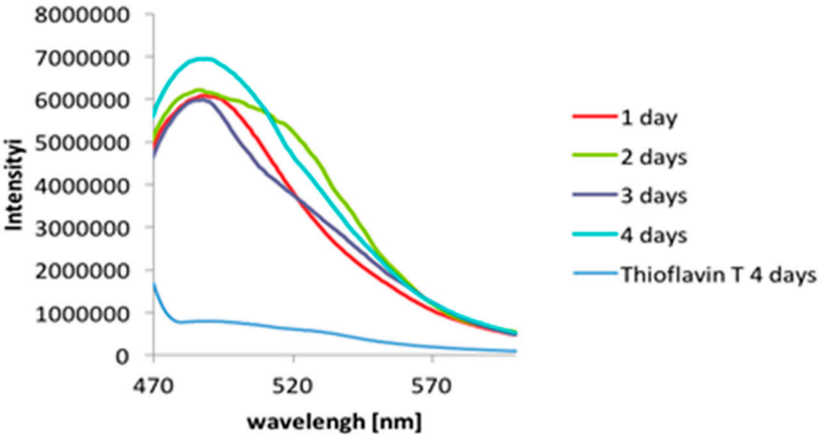

Fig. S43. Fluorescence intensity spectra of H_2N-Leu(*N*-Me)TyrGlnLeuGluAsnTyr-COOH (**1**).

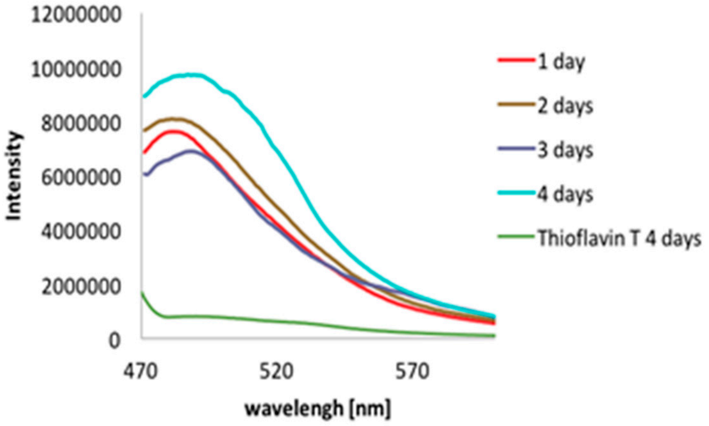

Fig. S44. Fluorescence intensity spectra of H_2N-LeuTyrGln(*N*-Me)LeuGluAsn-Tyr-COOH (**2**).

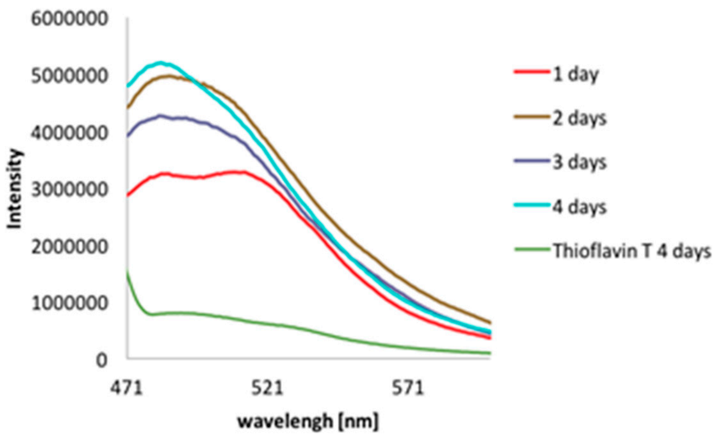

Fig. S45. Fluorescence intensity spectra of H_2N-Leu(*N*-Me)TyrGln(*N*-Me)LeuGluAsnTyr-COOH (**3**).

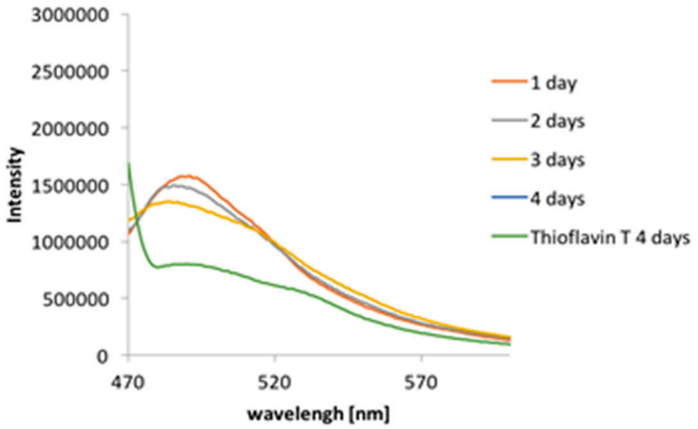

Fig. S46. Fluorescence intensity spectra of H_2N-ValGluAla(*N*-Me)LeuTyrLeu-COOH (**4**).

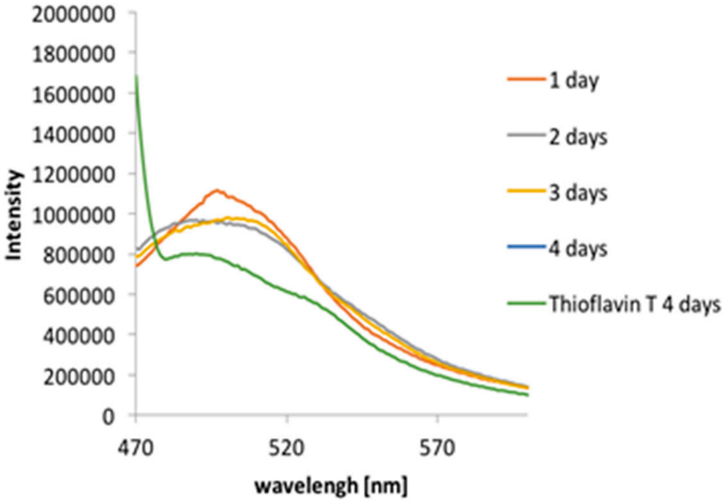

Fig. S47. Fluorescence intensity spectra of H_2N-ValGlu(*N*-Me)AlaLeuTyrLeu-COOH (**5**).

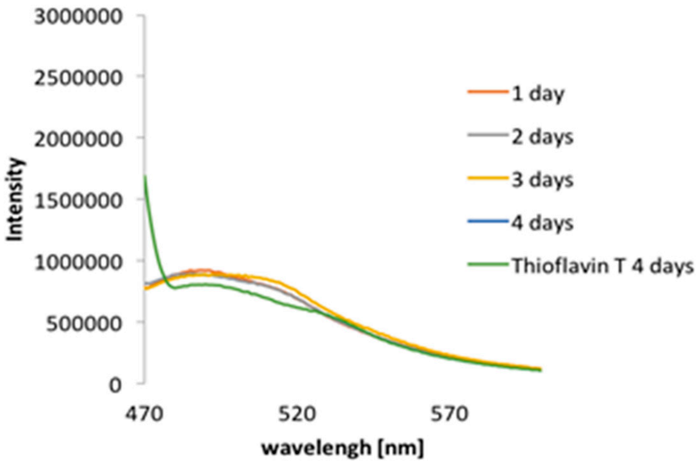

Fig. S48. Fluorescence intensity spectra of H_2N-ValGluAlaLeu(*N*-Me)TyrLeu-COOH (**6**).

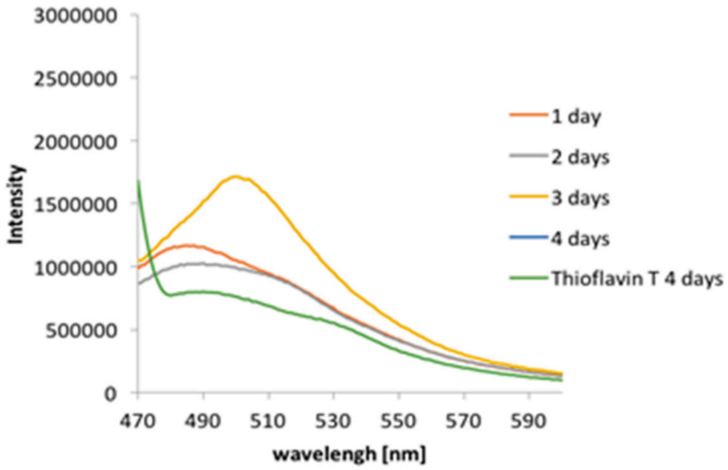

Fig. S49. Fluorescence intensity spectra of H_2N-ValGlu(*N*-Me)AlaLeu(*N*-Me)TyrLeu-COOH (**7**).

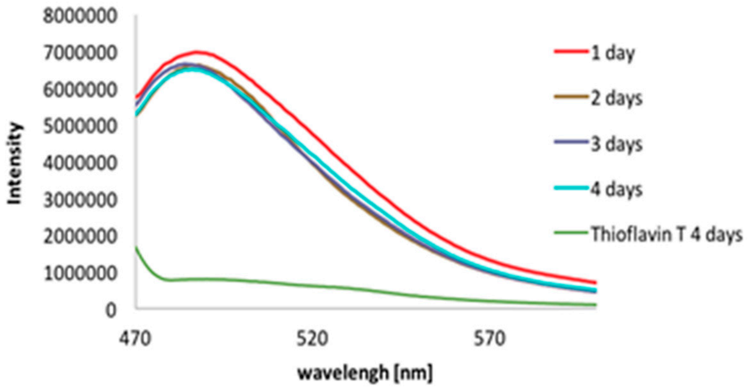

Fig. S50. Fluorescence intensity spectra of mixture of H_2N-Leu(*N*-Me)TyrGlnLeuGluAsnTyr-COOH (**1**) with hot spot A (1:1).

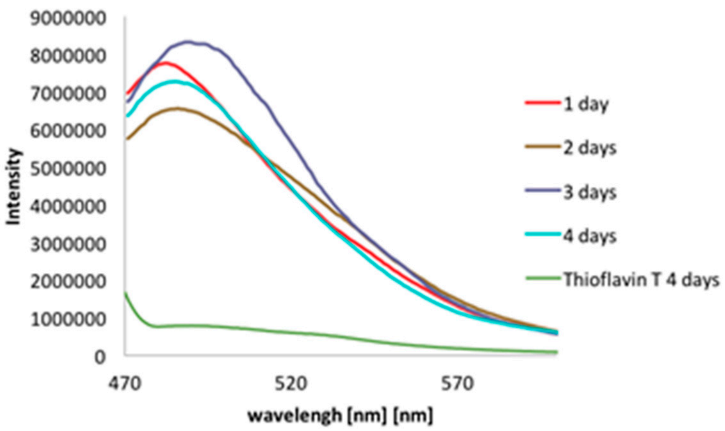

Fig. S51. Fluorescence intensity spectra of mixture of H_2N-LeuTyrGln(*N*-Me)LeuGluAsnTyr-COOH (**2**) with hot spot A (1:1).

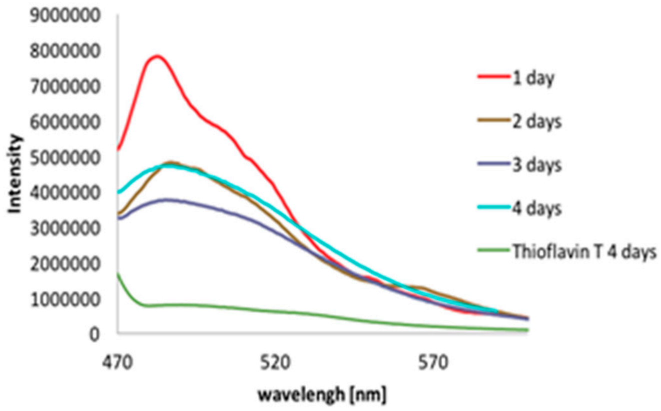

Fig. S52. Fluorescence intensity spectra of mixture of H_2N-Leu(*N*-Me)TyrGln(*N*-Me)LeuGlu-AsnTyr-COOH (**3**) with hot spot A (1:1).

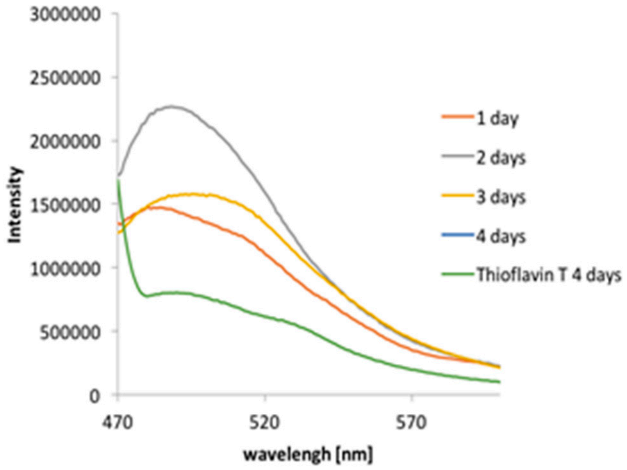

Fig. S53. Fluorescence intensity spectra of mixture of H_2N-ValGluAla(*N*-Me)LeuTyrLeu-COOH (**4**) with hot spot B (1:1).

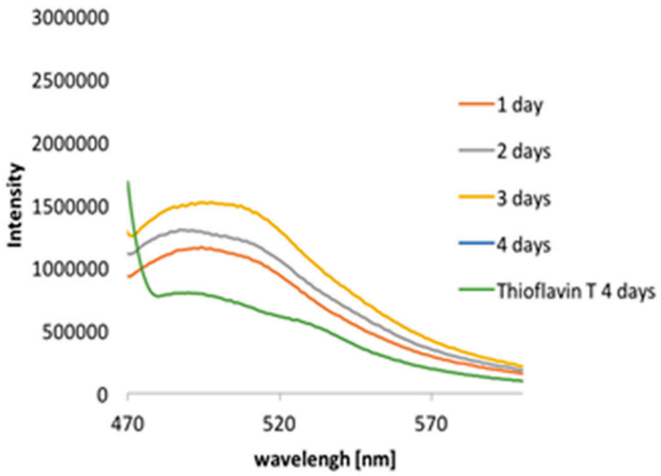

Fig. S54. Fluorescence intensity spectra of mixture of H_2N-ValGlu(*N*-Me)AlaLeuTyrLeu-COOH (**5**) with hot spot B (1:1).

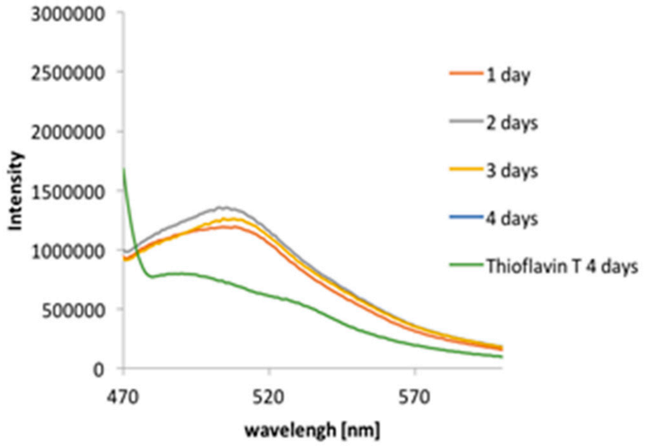

Fig. S55. Fluorescence intensity spectra of mixture of H_2N-ValGluAlaLeu(*N*-Me)TyrLeuCOOH (**6**) with hot spot B (1:1).

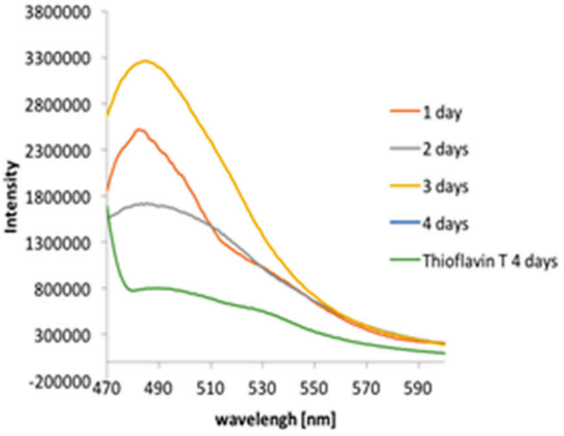

Fig. S56. Fluorescence intensity spectra of mixture of H_2N-ValGluAla(*N*-Me)Leu(*N*-Me)TyrLeu-COOH (**7**) with hot spot B (1:1).

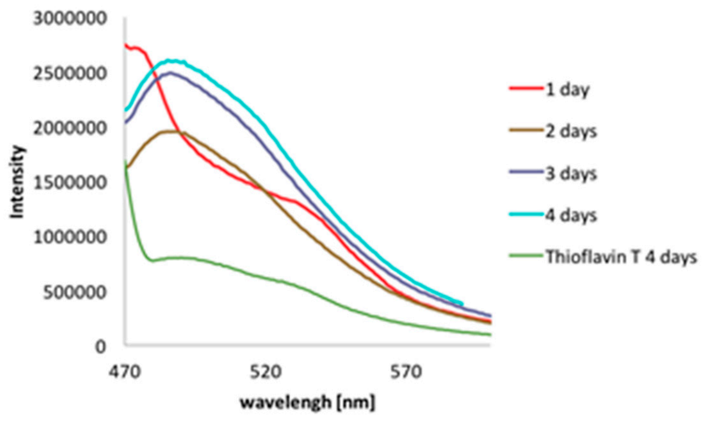

Fig. S57. Fluorescence intensity spectra of mixture of H_2N-Leu(*N*-Me)TyrGlnLeuGluAsnTyr-COOH (**1**) with hot spot A (2:1).

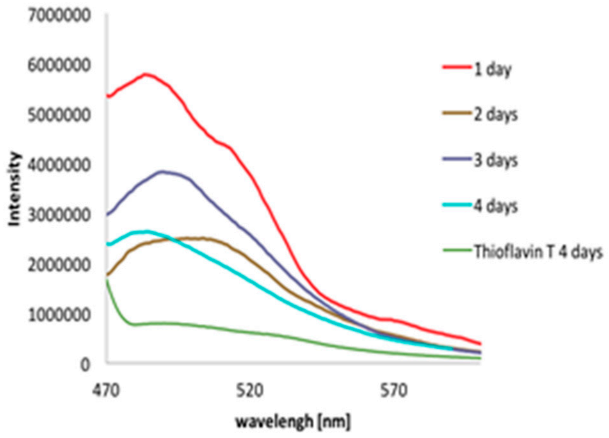

Fig. S58. Fluorescence intensity spectra of mixture of H_2N-LeuTyrGln(*N*-Me)LeuGluAsnTyr-COOH (**2**) with hot spot A (2:1).

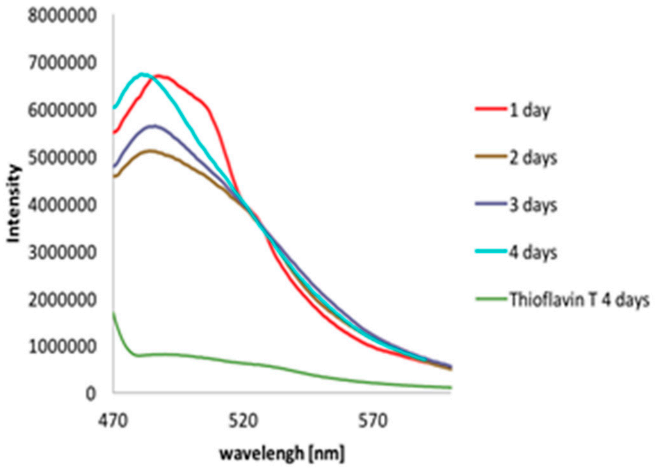

Fig. S59. Fluorescence intensity spectra of mixture of H_2N-Leu(*N*-Me)TyrGln(*N*-Me)LeuGluAsnTyr-COOH (**3**) with hot spot A (2:1).

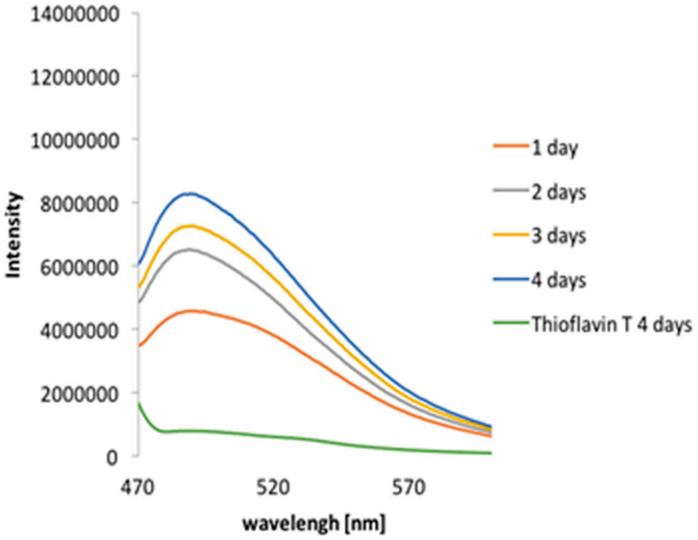

Fig. S60. Fluorescence intensity spectra of mixture of H_2N-ValGluAla(*N*-Me)LeuTyrLeuCOOH (**4**) with hot spot B (2:1).

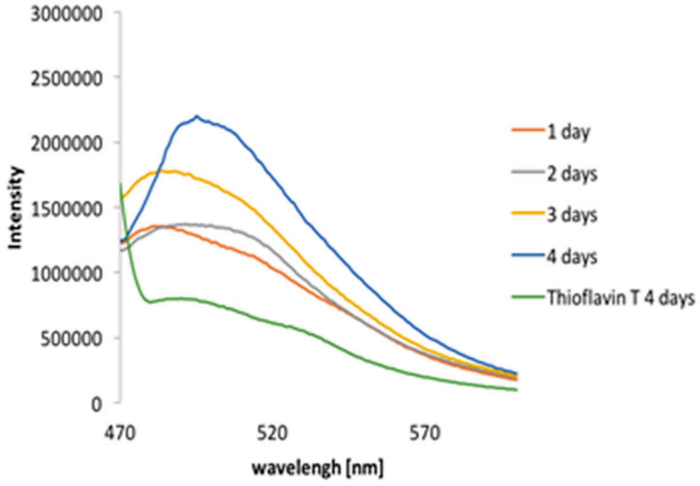

Fig. S61. Fluorescence intensity spectra of mixture of H_2N-ValGlu(*N*-Me)AlaLeuTyrLeuCOOH (**5**) with hot spot B (2:1).

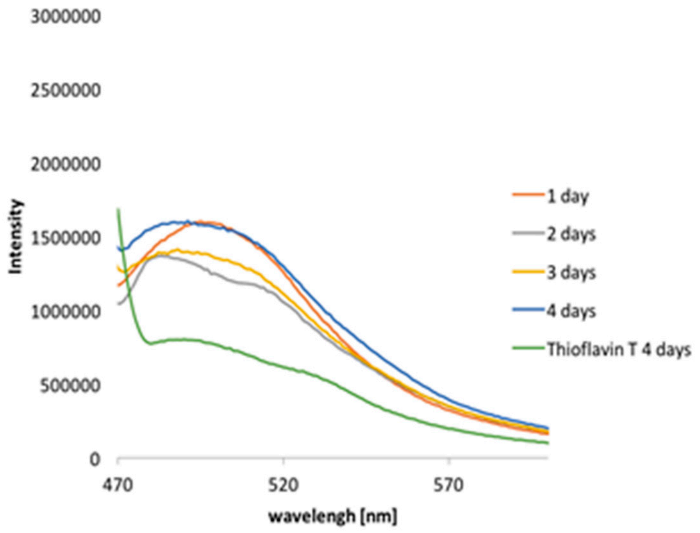

Fig. S62. Fluorescence intensity spectra of mixture of H_2N-ValGluAlaLeu(*N*-Me)TyrLeuCOOH (**6**) with hot spot B (2:1).

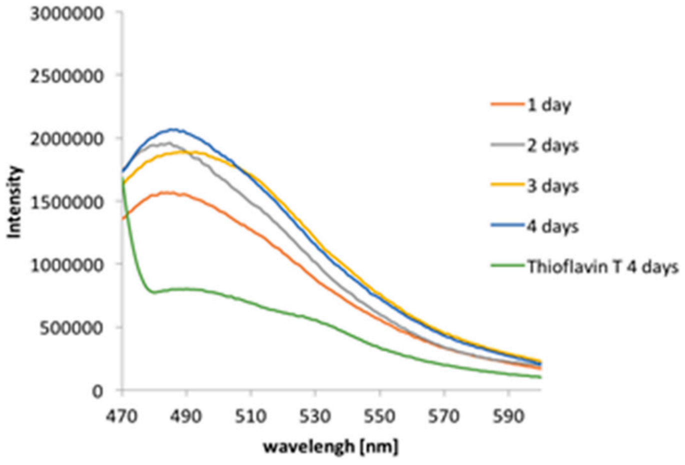

Fig. S63. Fluorescence intensity spectra of mixture of H_2N-ValGlu(*N*-Me)AlaLeu(*N*-Me)TyrLeu-COOH (**7**) with hot spot B (2:1).

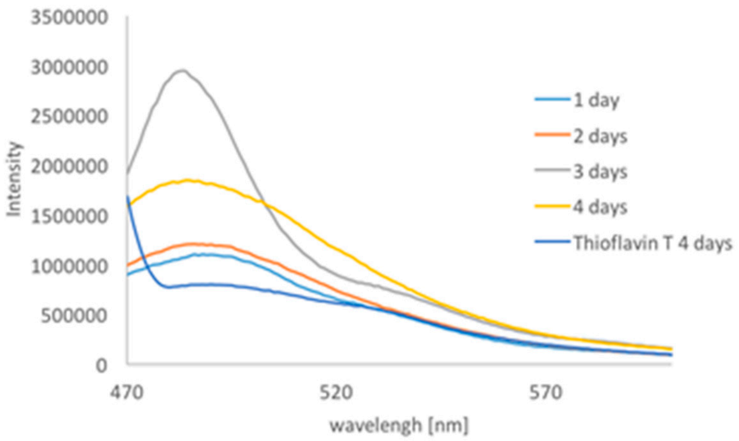

Fig. S64. Fluorescence intensity spectra of mixture of H_2N-Leu(*N*-Me)TyrGlnLeuGluAsnTyrCOOH (**1**) with insulin (1:1).

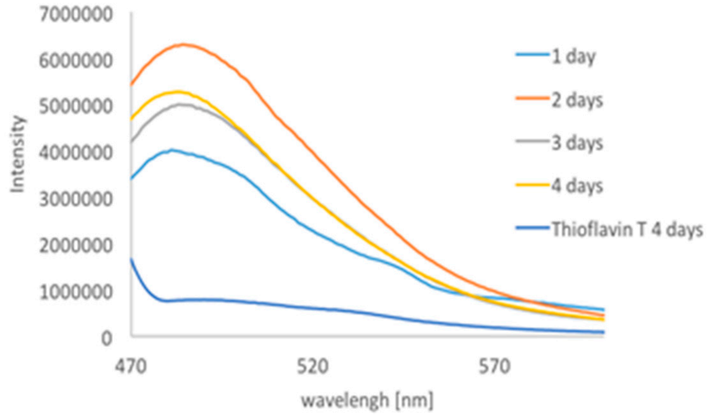

Fig. S65. Fluorescence intensity spectra of mixture of H_2N-LeuTyrGln(*N*-Me)LeuGluAsnTyr-COOH (**2**) with insulin (1:1).

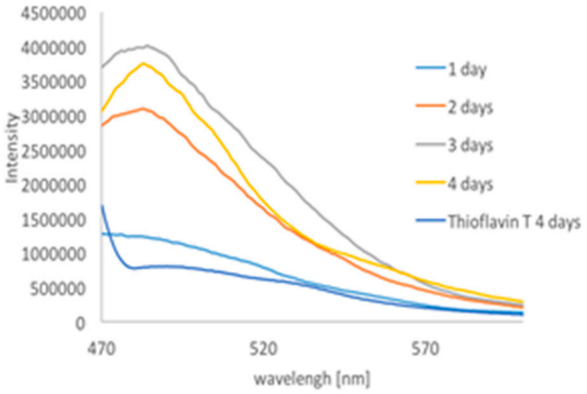

Fig. S66. Fluorescence intensity spectra of mixture of H_2N-Leu(*N*-Me)TyrGln(*N*-Me)LeuGluAsnTyr-COOH (**3**) with insulin (1:1).

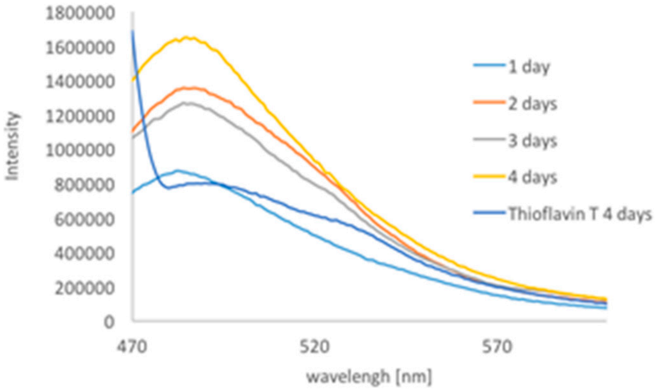

Fig. S67. Fluorescence intensity spectra of mixture of H_2N-ValGluAla(*N*-Me)LeuTyrLeu-COOH (**4**) with insulin (1:1).

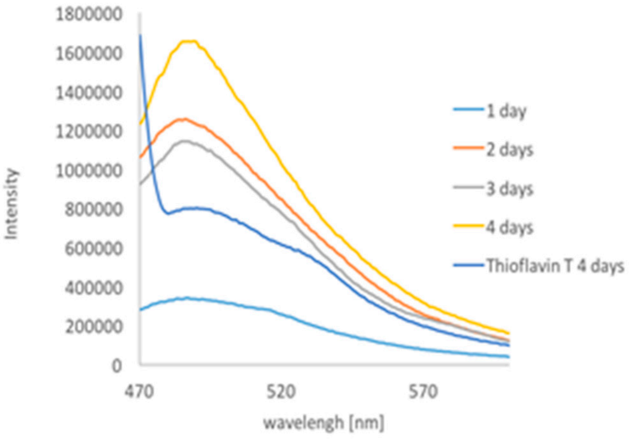

Fig. S68. Fluorescence intensity spectra of mixture of H_2N-ValGlu(*N*-Me)AlaLeuTyrLeu-COOH (**5**) with insulin (1:1).

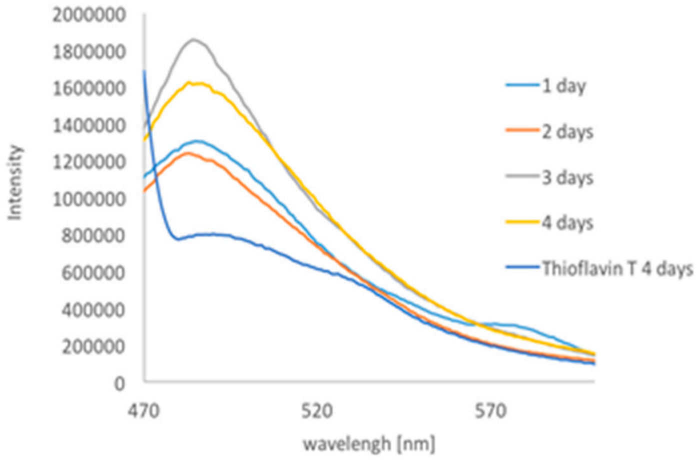

Fig. S69. Fluorescence intensity spectra of mixture of H_2N-ValGluAlaLeu(*N*-Me)TyrLeu-COOH (**6**) with insulin (1:1).

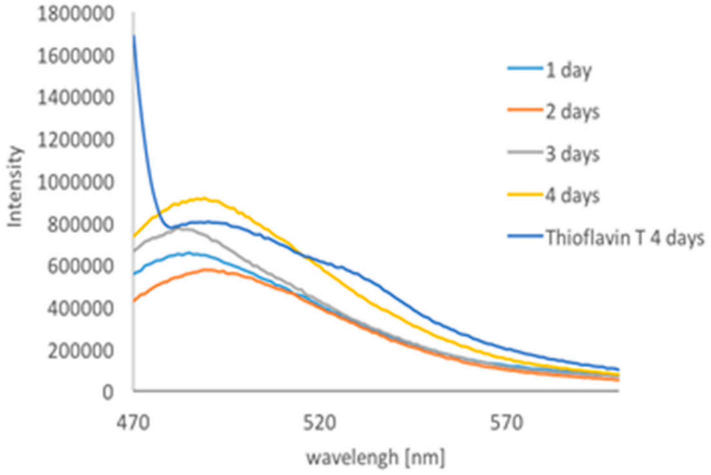

Fig. S70. Fluorescence intensity spectra of mixture of H_2N-ValGlu(*N*-Me)AlaLeu(*N*-Me)TyrLeu-COOH (**7**) with insulin (1:1).

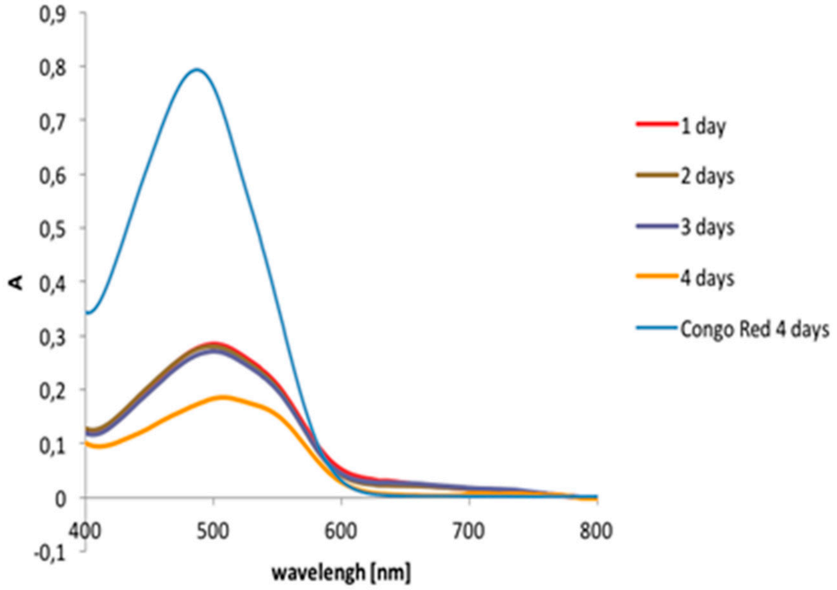

Fig. S71. UV spectra of hot spot A.

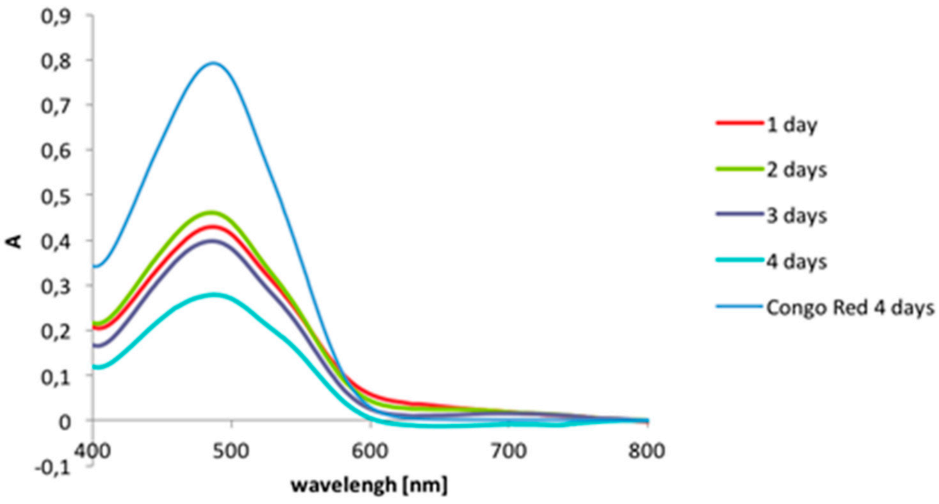

Fig. S72. UV spectra of hot spot B.

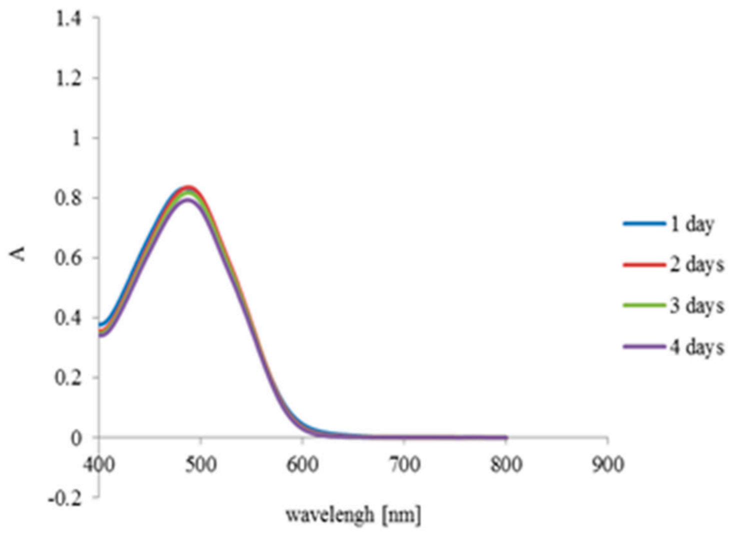

Fig. S73. UV spectra of Congo Red dye.

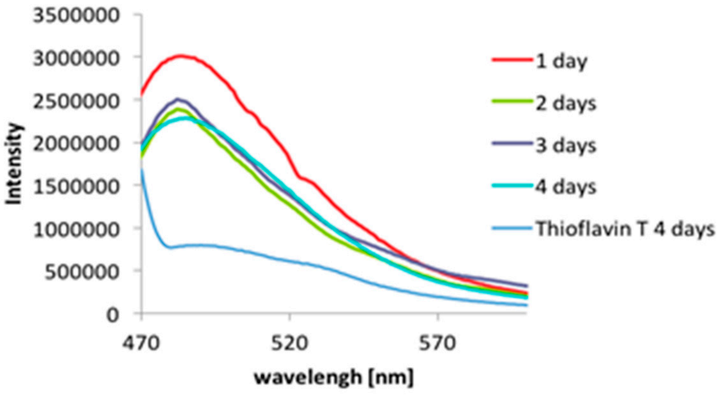

Fig. S74. Fluorescence intensity spectra of hot spot A.

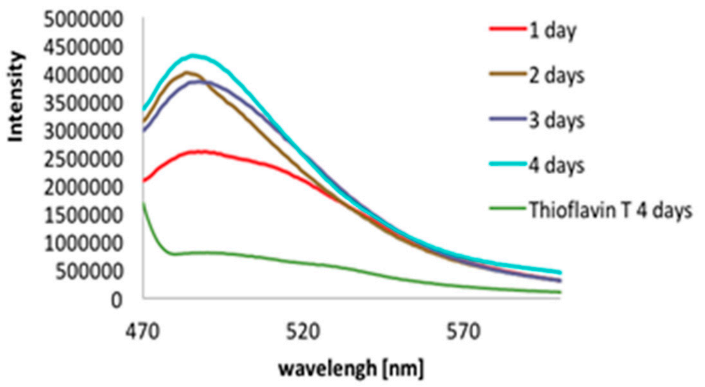

Fig. S75. Fluorescence spectra of hot spot B.
